# Genome-wide identification of *ZmSnRK2* genes and functional analysis of ZmSnRK2.10 in ABA signaling pathway in maize (*Zea mays* L)

**DOI:** 10.1186/s12870-021-03064-9

**Published:** 2021-07-01

**Authors:** Tiandan Long, Binjie Xu, Yufeng Hu, Yayun Wang, Changqing Mao, Yongbin Wang, Junjie Zhang, Hanmei Liu, Huanhuan Huang, Yinghong Liu, Guowu Yu, Chunzhao Zhao, Yangping Li, Yubi Huang

**Affiliations:** 1 State Key Laboratory of Crop Gene Exploration and Utilization in Southwest China, Chengdu, 611130 Sichuan China; 2 College of Agronomy, Sichuan Agricultural University, No.211 Huimin Rd., Wenjiang Dist, Chengdu, 611130 Sichuan China; 3 Triticeae Research Institute, Sichuan Agricultural University, Chengdu, 611130 Sichuan China; 4 College of Life Science, Sichuan Agricultural University, Ya’an, 625014 Sichuan China; 5 Maize Research Institute, Sichuan Agricultural University, Chengdu, 611130 Sichuan China; 6 Shanghai Center for Plant Stress Biology and CAS Center for Excellence in Molecular Plant Sciences, Chinese Academy of Sciences, Shanghai, 200032 China

**Keywords:** Maize, ABA, SnRK2 family, Evolution, Expression pattern, ZmSnRK2.10, Function analysis

## Abstract

**Background:**

Phytohormone abscisic acid (ABA) is involved in the regulation of a wide range of biological processes. In Arabidopsis, it has been well-known that SnRK2s are the central components of the ABA signaling pathway that control the balance between plant growth and stress response, but the functions of ZmSnRK2 in maize are rarely reported. Therefore, the study of *ZmSnRK2* is of great importance to understand the ABA signaling pathways in maize.

**Results:**

In this study, 14 *ZmSnRK2* genes were identified in the latest version of maize genome database. Phylogenetic analysis revealed that ZmSnRK2s are divided into three subclasses based on their diversity of C-terminal domains. The exon-intron structures, phylogenetic, synteny and collinearity analysis indicated that SnRK2s, especially the subclass III of SnRK2, are evolutionally conserved in maize, rice and Arabidopsis. Subcellular localization showed that ZmSnRK2 proteins are localized in the nucleus and cytoplasm. The RNA-Seq datasets and qRT-PCR analysis showed that *ZmSnRK2* genes exhibit spatial and temporal expression patterns during the growth and development of different maize tissues, and the transcript levels of some *ZmSnRK2* genes in kernel are significantly induced by ABA and sucrose treatment. In addition, we found that *ZmSnRK2.10*, which belongs to subclass III, is highly expressed in kernel and activated by ABA. Overexpression of *ZmSnRK2.10* partially rescued the ABA-insensitive phenotype of *snrk2.2/2.3* double and *snrk2.2/2.3/2.6* triple mutants and led to delaying plant flowering in Arabidopsis.

**Conclusion:**

The *SnRK2* gene family exhibits a high evolutionary conservation and has expanded with whole-genome duplication events in plants. The *ZmSnRK2s* expanded in maize with whole-genome and segmental duplication, not tandem duplication. The expression pattern analysis of *ZmSnRK2s* in maize offers important information to study their functions. Study of the functions of *ZmSnRK.10* in Arabidopsis suggests that the ABA-dependent members of *SnRK2s* are evolutionarily conserved in plants. Our study elucidated the structure and evolution of *SnRK2* genes in plants and provided a basis for the functional study of ZmSnRK2s protein in maize.

**Supplementary Information:**

The online version contains supplementary material available at 10.1186/s12870-021-03064-9.

## Background

Phytohormone abscisic acid (ABA) is widely involved in plant growth and development, responses to biotic and abiotic stresses, as well as crop grain filling and seed maturation processes [1, 2]. The core regulatory components of ABA signaling have been identified and studied well in Arabidopsis. ABA binds to the receptors pyrabactin resistance 1 (PYR1) and PYR1-like proteins (PYLs), which interact with and inhibit clade-A protein phosphatase type 2Cs (PP2Cs), leading to the release of SnRK2 (Sucrose non-fermenting protein kinase 2). The activated SnRK2s subsequently phosphorylate downstream target proteins such as ABA-responsive element-binding factors (ABFs) to mediate various ABA responses [3–7]. SnRK2s are crucial for ABA signaling because ABA-mediated regulation of various physiological processes is attributed to the SnRK2s-mediated phosphorylation of different downstream targets.

The first *SnRK2* gene, named *PKABA1*, was cloned and characterized in wheat. PKABA1 is involved in the phosphorylation of downstream ABA responsive transcription factor TaABF1 [8]. So far, the SnRK2 proteins have been identified and characterized in many species, including 10 members (SnRK2.1 to − 2.10) in Arabidopsis [9] and 10 (SAPK1 to − 10) in rice [10]. SnRK2 family can be divided into three subclasses (subclass I, II and III) based on the sequence of amino acids at the C-terminal domain [9]. Analysis of the kinase activity of SnRK2s in Arabidopsis and rice indicates that each subclass of SnRK2 has a discrepant activation pattern in response to ABA and osmotic stress. Subclass I SnRK2s are quickly and strongly activated by osmotic stress, while the ABA-independent subclass II and III SnRK2s are activated by both ABA and osmotic stress. Specifically, subclass II SnRK2s are weakly activated by ABA, and subclass III SnRK2s are rapidly and strongly activated by ABA. Therefore, subclass III SnRK2s are widely considered as key regulators in ABA signaling pathway [10–14]. In Arabidopsis, subclass III SnRK2s are encoded by three genes, *SnRK2.2*, *SnRK2.3*, and *SnRK2.6* which are quickly activated by ABA treatment [11, 15]. *snrk2.2/2.3/2.6* triple mutant exhibits a small plant size and is insensitive to ABA in terms of seed germination and seeding growth [16]. Although *SnRK2.2*, *SnRK2.3*, and *SnRK2.6* function redundantly in ABA-mediated regulation of plant growth and stress responses, there also exhibit distinct functions in some aspects. *SnRK2.6* is mainly required for ABA-induced stomatal closure, and therefore *snrk2.6* single mutant leads to severe water loss in Arabidopsis leaves [17, 18]. *SnRK2.2* and *SnRK2.3* are particularly involved in the regulation of seed germination and seedling growth. *snrk2.2/2.3* double mutant can germinate in the presence of extremely high ABA concentration [19]. In rice, subclass III SnRK2s include three genes, *SAPK8*, *SAPK9*, and *SAPK10* which are homologous to Arabidopsis *SnRK2.2*, *SnRK2.3*, and *SnRK2.6* respectively. These three SnRK2 proteins are also activated by ABA [10, 20]. Compared with Wild type (WT), transgenic rice lines overexpressing *SAPK8*, *SAPK9*, or *SAPK10* show delayed germination and seedling growth [21], suggesting that the subclass III SnRK2s in rice is also involved in the regulation of seeds germination.

Maize (*Zea mays* L.) not only serves as grain and forage crop but also is widely used as main raw material in many fields such as food and bioenergy industries. In maize, ABA coupled with the VIVIPAROUS1 (VP1) transcription factor plays an essential role in kernel development and maturation [22, 23]. In addition, ABA is also a key factor in the regulation of programmed cell death during maize endosperm development [24, 25]. Here, we conducted genome-wide identification of *ZmSnRK2* gene family in maize, and the gene structure, chromosomal location, phylogenetic relationship, synteny, and expression profiles of these *ZmSnRK2s* were investigated. Moreover, we found that *ZmSnRK2.10* gene, which belongs to subclass III, was activated by ABA in maize. Overexpression of *ZmSnRK2.10* could partially complement the phenotype of *Atsnrk2.2/2.3*, *Atsnrk2.2/2.3/2.6* mutants and delay the flowering time in Arabidopsis*.* This study will enhance our understanding of the *SnRK2* gene family and provide insights into the functional diversity of *SnRK2* genes in maize.

## Results

### Genome-wide identification and evolutionary analysis of *SnRK2* genes in maize

To identify possible SnRK2 homologs in MaizeGDB database (https://maizegdb.org/), we constructed the HMM (Hidden Markov Model) of SnRK2s based on their protein sequences in *Arabidopsis thaliana* and *Oryza sativa*, and used it for BLASTP analysis. A total of 14 candidate *ZmSnRK2* genes were identified and characterized (Additional file 1: Table S1), among which 10 *ZmSnRK2* genes have been cloned and reported previously [26], we cloned the *ZnSnRK2.9* gene and the remaining three newly identified *ZmSnRK2* genes were designated *ZmSnRK2.12* to *ZmSnRK2.14*. To characterize the properties of the identified SnRK2s in maize, the protein sequences of ZmSnRK2s were analyzed by ExPASy (https://web.expasy.org/compute_pi/). As shown in Table S1, the protein lengths of ZmSnRK2s range from 333 amino acids (ZmSnRK2.3) to 366 amino acids (ZmSnRK2.8 and ZmSnRK2.12) and the molecular weights range from 37.84 kDa (ZmSnRK2.3) to 42.84 kDa (ZmSnRK2.5). The ZmSnRK2 proteins were acidic with predicted pI values varying from 4.57 to 6.73.

The full-length of 14 ZmSnRK2 proteins downloaded from Gramene (http://ensembl.gramene.org/) were clustered by clustalw1.83, and then a neighbor-joining (NJ) phylogenetic tree was constructed (Additional file 1: Fig. S1A). Comparative analysis showed that *ZmSnRK2s* were clustered into three subclasses, in which *ZmSnRK2.12* with *ZmSnRK2.8*, *ZmSnRK2.9* and *ZmSnRK2.10* was classified as subclass III, while *ZmSnRK2.13* and *ZmSnRK2.14* with *ZmSnRK2.4*, *ZmSnRK2.5*, *ZmSnRK2.6*, *ZmSnRK2.7* and *ZmSnRK2.11* were clustered into subclass I. Based on previous reports [26, 27], *ZmSnRK2.3* with *ZmSnRK2.1* and *ZmSnRK2.2* were classified as subclass II. The conserved motifs in ZmSnRK2 proteins were analyzed by MEME (http://meme-suite.org/), all the ZmSnRK2 proteins were identified to contain six large conserved motifs (Additional file 1: Fig. S1A). Among these proteins, the location of the conserved motifs in subclass III proteins showed a high similarity, while the other 10 ZmSnRK2s (subclass I and subclass II) exhibited a similar pattern of motifs distribution. Protein sequences alignment indicated that the ZmSnRK2s were evolutionarily conserved, and the variations in ZmSnRK2 proteins were mainly identified at C-terminal regions (Additional file 1: Fig. S1B).

**Fig. 1:**
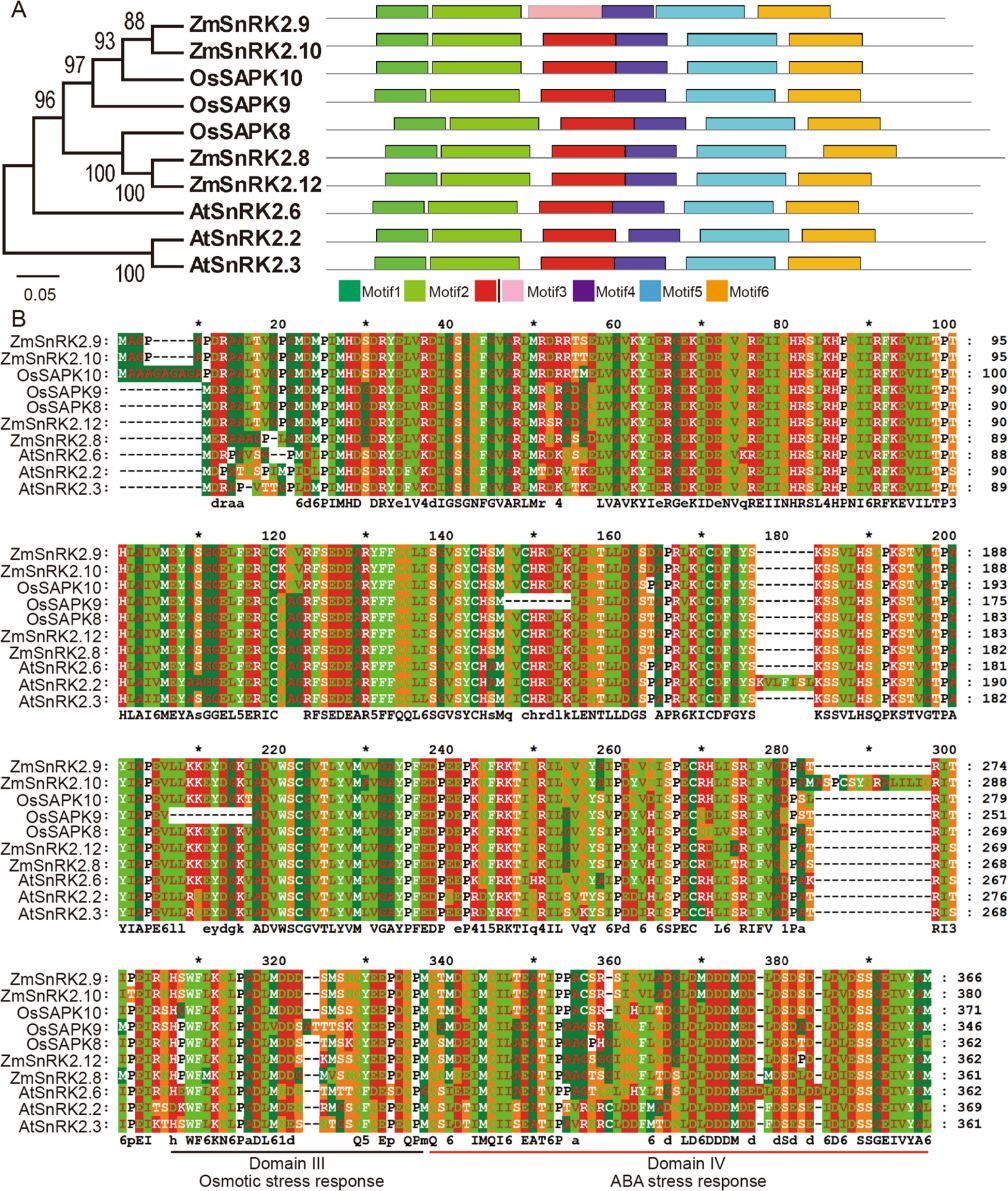
Phylogenetic relationships, conserved motifs, and amino acid sequence alignment of subclass III SnRK2s in maize, rice and Arabidopsis. (**A**) Phylogenetic tree and conserved motifs analysis of subclass III SnRK2s family in maize, rice and Arabidopsis. (**B**) Amino acid sequence alignment of the subclass III SnRK2 proteins in maize, rice and Arabidopsis. Domain III is responsible for osmotic stress response, and domain IV functions in ABA stress response

To investigate the evolutionary features of identified *ZmSnRK2* genes in maize with that in other plants, phylogenetic and conserved motifs analysis were performed based on the amino acid sequences of all SnRK2 proteins from *Zea mays*, *Arabidopsis thaliana* and *Oryza sativa.* All these SnRK2s were clustered into three subclasses and contain six large conserved motifs too (Additional file 1: Fig. S2A, Fig. S2B). It is worth noting that *ZmSnRK2.8*/*2.9*/*2.10*/*2.12* belong to the same branch with *SnRK2.2*/*2.3*/*2.6* in Arabidopsis and *SAPK8*/*9*/*10* in rice and exhibited a similar pattern of conserved motifs distribution (Fig. 1A). *SnRK2.2*/*2.3*/*2.6* play a crucial role in responding to ABA signal pathway in Arabidopsis [28], which may suggest these four members of subclass III play an important function in response to ABA signal in maize. The other two newly identified *ZmSnRK2* genes (*ZmSnRK2.13* and *ZmSnRK2.14*) were clustered with *AtSnRK2s* in Arabidopsis that are particularly involved in response to osmosis stress [16], which suggested a possible role of these two *ZmSnRK2s* in the regulation of osmotic stress in maize. The difference between subclass III with subclass I and subclass II was that aspartic acid (short for D) was enriched in the C-terminal regions of subclass III proteins, while glutamate (short for E) was enriched in the C-terminal regions of subclass I and II proteins (Fig. 1B and Additional file 1: Fig. S2C).

### Genes structure, chromosomal localization and synteny analysis of *ZmSnRK2* genes in *Zea mays*

**Fig. 2:**
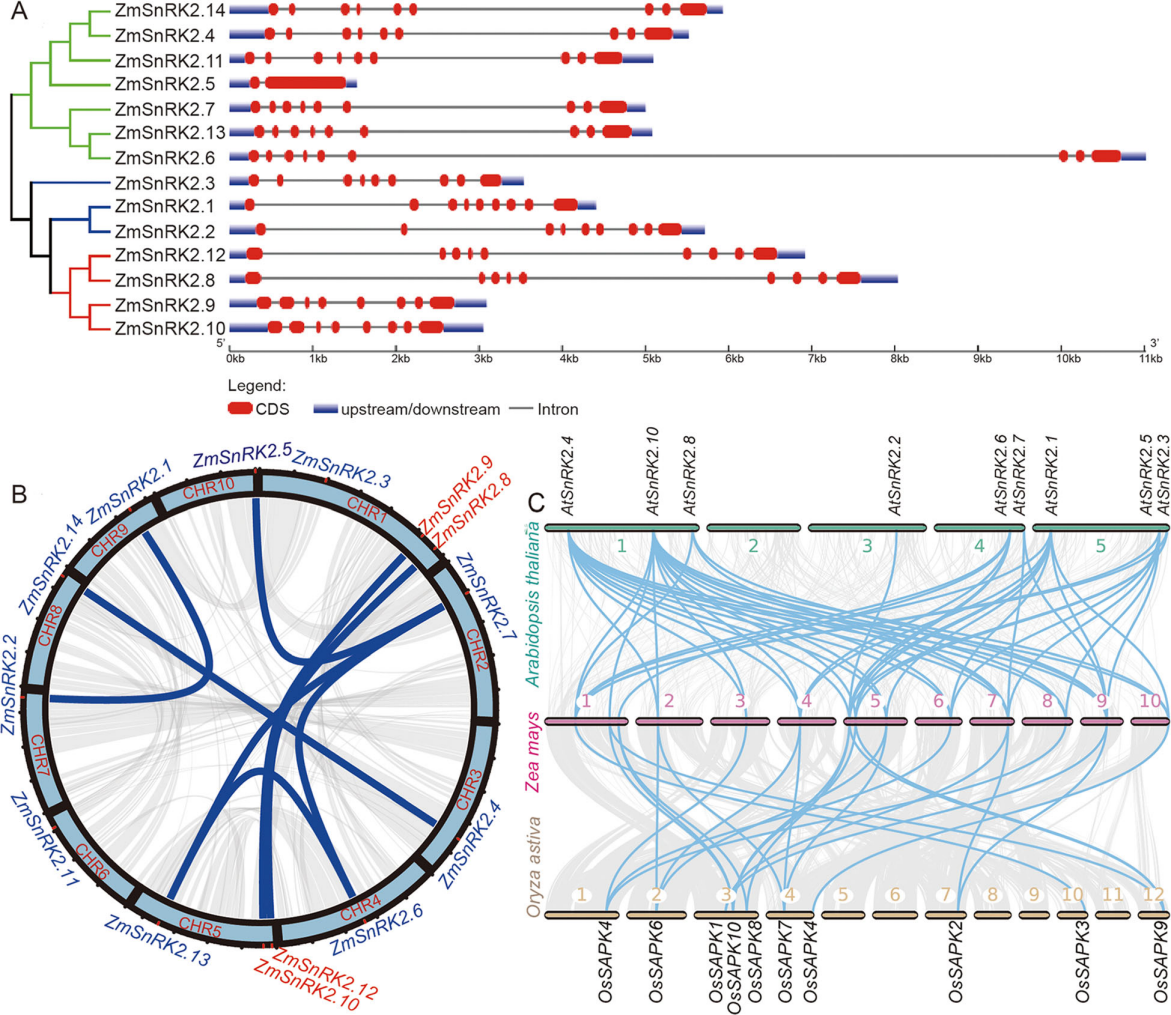
Distribution and gene duplications of *SnRK2s*. (**A**) The exon-intron structure of *ZmSnRK2s*. The exons, introns, and untranslated regions are indicated by red boxes, black lines and blue boxes, respectively. (**B**) Synteny and collinearity analysis of *SnRK2s* in maize. The positions of *SnRK2* genes were depicted on the chromosomes of *Zea mays.* The Arabic numerals in bars represent different chromosome respectively. Subclass III SnRK2 genes are colored with red. (**C**) Synteny and collinearity analysis of *SnRK2* genes among maize, Arabidopsis, and rice. The WGD (whole-genome duplication) or segmental duplication is linked by green lines, and gray lines in the background represent the collinear blocks among different chromosomes. These pictures of B and C were drawn by using Circos

To explore the structural diversity of the *ZmSnRK2* members, the intron-exon organization of each *ZmSnRK2* gene was analyzed by comparing the cDNA sequences with the corresponding genomic DNA sequences. As show in Fig. 2A, most of *ZmSnRK2* genes contain nine exons and eight introns, whereas *ZmSnRK2.5* only consists of a single intron. Compared with other *ZmSnRK2* genes, *ZmSnRK2.6* contains a long intron in its gene structure. Notably, three members (*ZmSnRK2.1*, *ZmSnRK2.2* and *ZmSnRK2.3*) clustered in subclass I share a high similarity in gene structure. For four *ZmSnRK2* members (*ZmSnRK2.8*, *ZmSnRK2.9*, *ZmSnRK2.10*, and *ZmSnRK2.12*) clustered in subclass III, *ZmSnRK2.8* and *ZmSnRK2.12* share a high similarity in gene structure, as well as *ZmSnRK2.9* and *ZmSnRK2.10*. Besides, *ZmSnRK2.14, ZmSnRK2.11* and *ZmSnRK2.4* share a high similarity in gene structure, as well as *ZmSnRK2.13* and *ZmSnRK2.7.*

Combining the information of the highly conserved motifs, phylogenetic and gene structures analysis of *ZmSnRK2s*, we speculated that the expansion of *ZmSnRK2s* in maize tended to be tandem or segmental duplication events. Firstly, the physical locations of the *ZmSnRK2* genes on chromosomes were analyzed based on the genome database. As the Fig. 2B shown that all the identified *ZmSnRK2* genes could be mapped on maize chromosomes from 1 to 10, with each 3 on chromosome 1 and 5. Interestingly, four *ZmSnRK2* members clustered in subclass III were located at the distal side of either chromosome 1 or chromosome 5. Moreover, *ZmSnRK2.8* and *ZmSnRK2.9* are closely associated on chromosome 1, while *ZmSnRK2.10* and *ZmSnRK2.12* are closely associated on chromosome 5. In addition, we identified many segmental duplication events but not tandem duplication events among *SnRK2s* in maize by using MCScanX program. Furthermore, synteny and collinear relationships among *Zea mays*, *Arabidopsis thaliana* and *Oryza sativa* were analyzed to investigate potential evolutionary events of *SnRK2s* in plants. Each member of *SnRK2* in maize has one or more orthologous genes in Arabidopsis and rice, and this collinearity relationship occurs in the same subclass (Fig. 2C), suggesting that the duplication events of *SnRK2s* have occurred in plants before the differentiation of maize, rice, and Arabidopsis. Together, these results indicated that the expansion of *SnRK2* genes family in maize mainly because of segmental duplication.

### Expression pattern analysis of *ZmSnRK2* genes in *Zea mays*

**Fig. 3:**
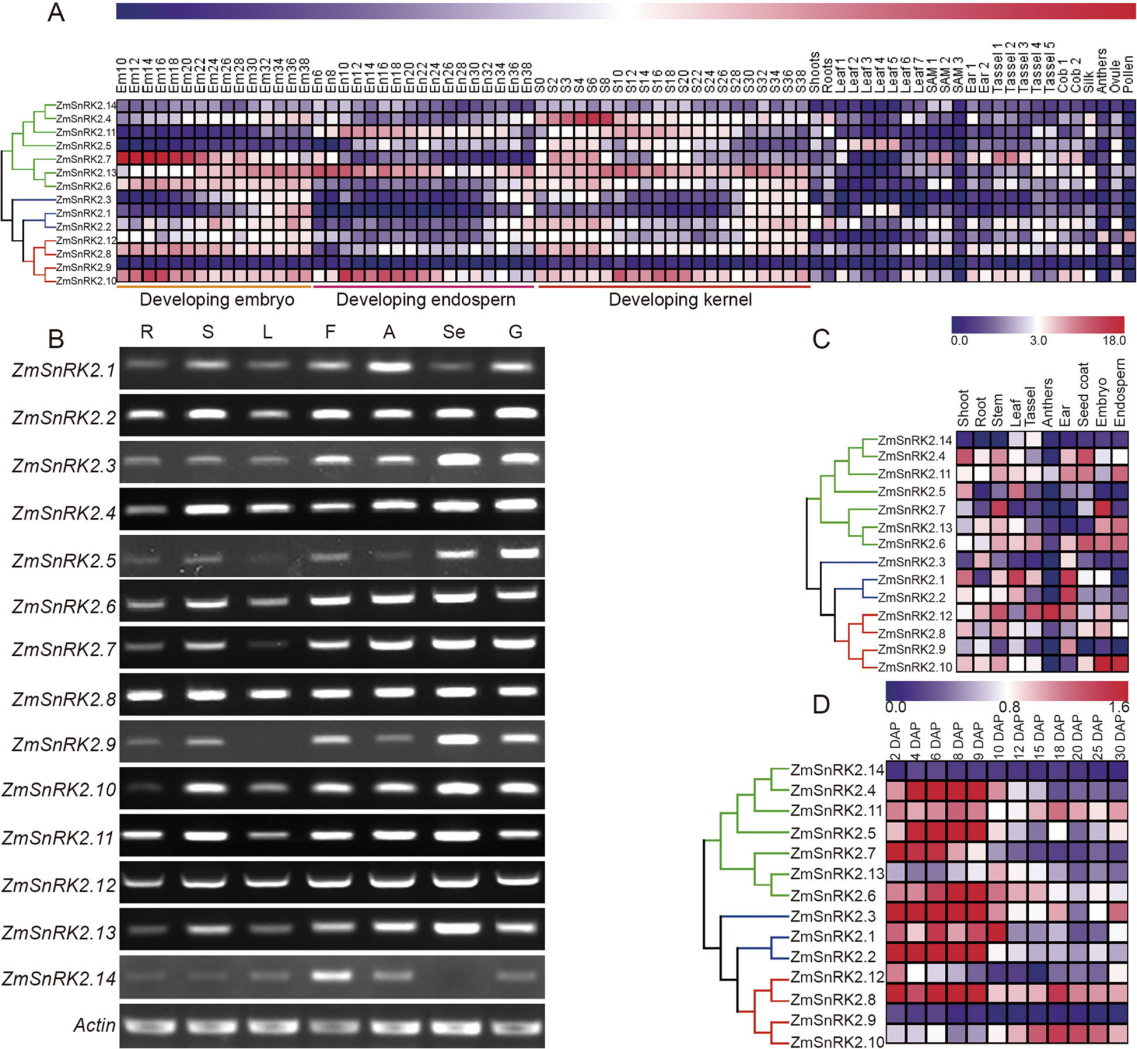
The expression profiling of *ZmSnRK2s* in maize. (**A**) The heatmap was generated based on the released RNA-seq data download from public database (http://www.ncbi.nlm. nih.gov/sra) under accession number SRP037559. The color scale is shown at the upper of the figure. Higher expression levels are shown in red, and lower in blue. The map was made by Mev 4.9. (**B**) Semi-quantitative RT-PCR analysis was conducted to analyze the expression of 14 *ZmSnRK2* genes in root (R), stem (S), leaf (L), flower (F), anther (A), Seed (Se), and Germinated Seed (G). (**C**) The heatmap indicates the relative expression of 14 *ZmSnRK2* genes in shoot, root, stem, leaf, tassel, anthers, seed coat (15 DAP), embryo (15 DAP), and endosperm (15 DAP) based on qRT-PCR data. (**D**) The heatmap indicates the expression of *ZmSnRK2* genes in the developing maize seed based on qRT–PCR data. S2-S30: seeds (2 DAP)-seeds (30 DAP), (DAP: days after pollination)

**Fig. 4:**
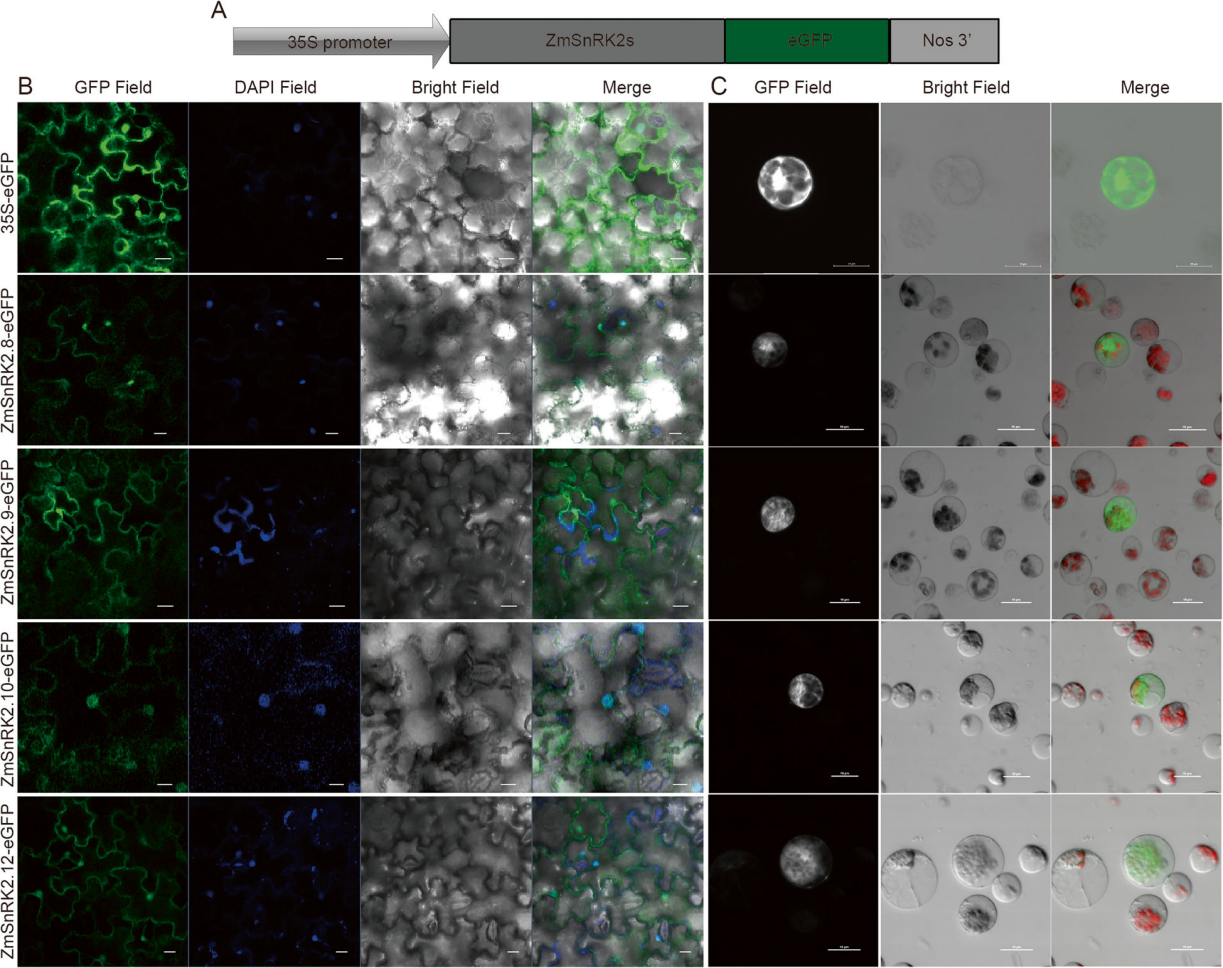
Subcellular localization of subclass III SnRK2 proteins. (**A**) Schematic illustration of the constructs. (**B**) The construct containing ZmSnRK2s:GFP fusion protein vector into the *Agrobacterium tumefaciens* strain GV3101 and used to infect *Nicotiana benthamiana* leaf cell. The bars, 50 μm. (**C**) The ZmSnRK2s:GFP fusion protein was transformed into maize leaf protoplasts respectively. The bars, 10 μm. All fluorescence signals were detected using the Confocal microscope. *35S-eGFP* was used as the control. Merged, individual images of GFP and bright field images of onion epidermal cells were shown. GFP, green fluorescent protein. The localization of the nuclei was indicated by DAPI (6-diamidino-2-phenylindole) staining

To explore the biological functions of *ZmSnRK2* genes, we first analyzed the expression profile of *ZmSnRK2* genes in 78 samples. These samples were derived from different tissues at different developmental stages of maize and the transcriptome data in these samples were downloaded from NCBI database (http://www.ncbi.nlm.nih.gov/sra) [29]. The *ZmSnRK2* genes showed different spatial and temporal expression patterns in maize (Fig. 3A). For the members of subclass III, the expression level of *ZmSnRK2.8* was relatively stable at each stage of the kernel development, but was significantly higher in the embryo than that of the endosperm. The expression level of *ZmSnRK2.9* was low in all samples. Notably, *ZmSnRK2.10* was highly expressed during the development of kernel, especially in the 10 DAP (days after pollination) and 24 DAP of kernel development, the stage of which is very important for grain filling and seed maturation. *ZmSnRK2.12* was the only gene that showed a high expression level in pollen. Besides, the expression level of *ZmSnRK2.14* was relative lower in almost all tissues, whereas the *ZmSnRK2.13* was highly expressed in seeds (Fig. 3A, B). The expression of *ZmSnRK2* genes in several maize tissues or during kernel development was examined by semi-quantitative RT-PCR (sqRT-PCR) or qRT-PCR analysis, and results obtained from qRT-PCR analysis were almost consistent with those obtained from the public database (Figs. 3B-4D). ABA and sucrose act as signal molecules to regulate the expression of genes involved in metabolism during grain filling [2, 30]. We then analyzed the expression changes of the *ZmSnRK2* genes in maize kernel under the treatment of ABA and sucrose, and the result indicated that the *ZmSnRK2* genes were differentially expressed after ABA or sucrose treatment (Additional file 1: Fig. S3). For example, the expression levels of *ZmSnRK2.1/2.3/2.6/2.9/2.10* were upregulated under ABA treatment, whereas the transcript levels of *ZmSnRK2.8/2.9/2.10/1.12*, members of subclass III, were increased after sucrose treatment. Besides, the expression level of *ZmSnRK2.13* was downregulated under both ABA and sucrose treatment.

### ZmSnRK2 proteins are localized in cytoplasm and nucleus

The cytoplasmic and nuclear localization of ZmSnRK2.1 to ZmSnRK2.11 (except ZmSnRK2.9) proteins in *Nicotiana benthamiana* leaves have been reported [31]. Here, to determine the subcellular localization of all ZmSnRK2 proteins accurately, we generated *ZmSnRK2s-GFP* fusion constructs driven by the cauliflower mosaic virus (CaMV) 35S promoter, and transiently transformed them to both maize protoplast and *Nicotiana benthamiana* leaves. A strong green fluorescence signal was observed in the nucleus and cytoplasm of the maize protoplasts or *Nicotiana benthamiana* leaves cells transformed with *ZmSnRK2s-GFP*, while the free GFP protein was detected in the cytoplasm, plasma membrane and nuclei (Fig. 4 and Additional file 1: Fig. S4).

### The kinase activity of ZmSnRK2.10 protein is activated by ABA

**Fig. 5:**
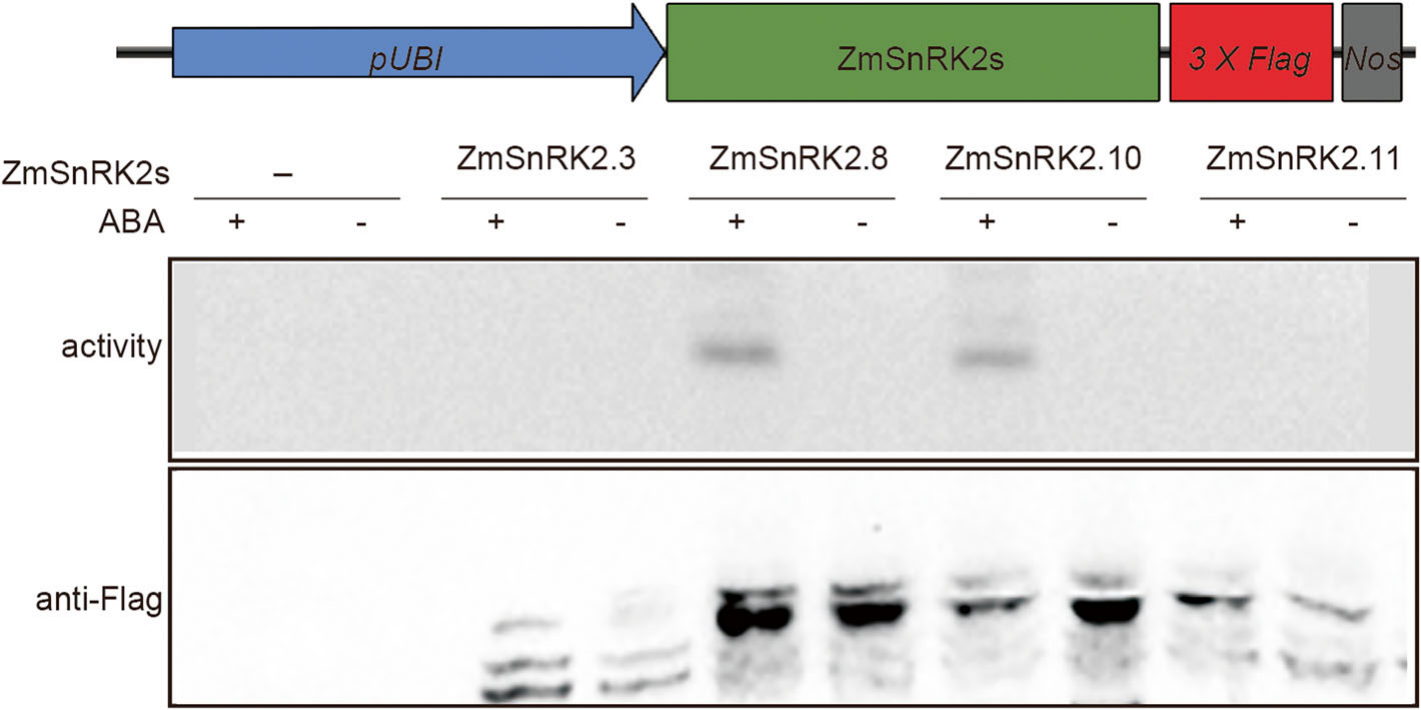
The ZmSnRK2.10 kinase activity is activated by ABA. Mazie leaf protoplasts were transiently transformed with empty vector or the constructs expressing the indicated *SnRK2s*. The SnRK2 proteins were immunoprecipitated by using anti-Flag antibody (lower panel) and the kinase activity of SnRK2s was analyzed by in vitro kinase assay using MBP as a substrate (upper panel)

Considering that the *ZmSnRK2.10*, a subclass III SnRK2s, was highly expressed during kernel development and was induced by ABA, we investigated whether the ZmSnRK2.10 protein kinases is activated by ABA and whether it is directly involved in ABA signaling pathway. The luciferase reporter gene driven by the ABA-responsive *RD29B* promoter was used to test the roles of *ZmSnRK2.10* in ABA signaling pathway. Four selected *ZmSnRK2s* members, including *ZmSnRK2.8* (*ZmOST1*) that is activated by ABA and weakly mediates the closing of stomata in maize [32], *ZmSnRK2.10*, *ZmSnRK2.11* (member of subclass I) and *ZmSnRK2.3* (member of subclass II) were tested. The *ZmSnRK2* genes and reporter gene were co-transformed into maize leaf protoplasts. The result showed that the protoplast expressing *ZmSnRK2.8* or *ZmSnRK2.10* exhibited a higher *RD29B*-LUC expression than that expressing *ZmSnRK2.3* or *ZmSnRK2.11*. Similar to *ZmSnRK2.8*, the up-regulation of *RD29B-LUC* expression by *ZmSnRK2.10* was extremely enhanced by ABA treatment (Additional file 1: Fig. S5). To further confirm that the ZmSnRK2.10 protein kinase was directly activated by ABA, immunoprecipitation-kinase (IP-kinase) assay was performed using MBP as substrates to investigate the kinase activity of with or without ABA treatment. After immunoprecipitation of ZmSnRK2 proteins by anti-Flag antibody, in vitro kinase assay was performed. The result showed that none of these ZmSnRK2s exhibited kinase activity without ABA treatment. However, the kinase activities of ZmSnRK2.8 and ZmSnRK2.10, but not ZmSnRK2.3 and ZmSnRK2.11, were induced after ABA treatment (Fig. 5). These results indicated that both ZmSnRK2.8 and ZmSnRK2.10 are activated by ABA and are involved in the regulation of ABA-responsive genes expression.

### Overexpression of *ZmSnRK2.10* partially rescues the ABA-insensitive phenotype of *snrk2.2/2.3* and *snrk2.2/2.3/2.6* mutants

**Fig. 6:**
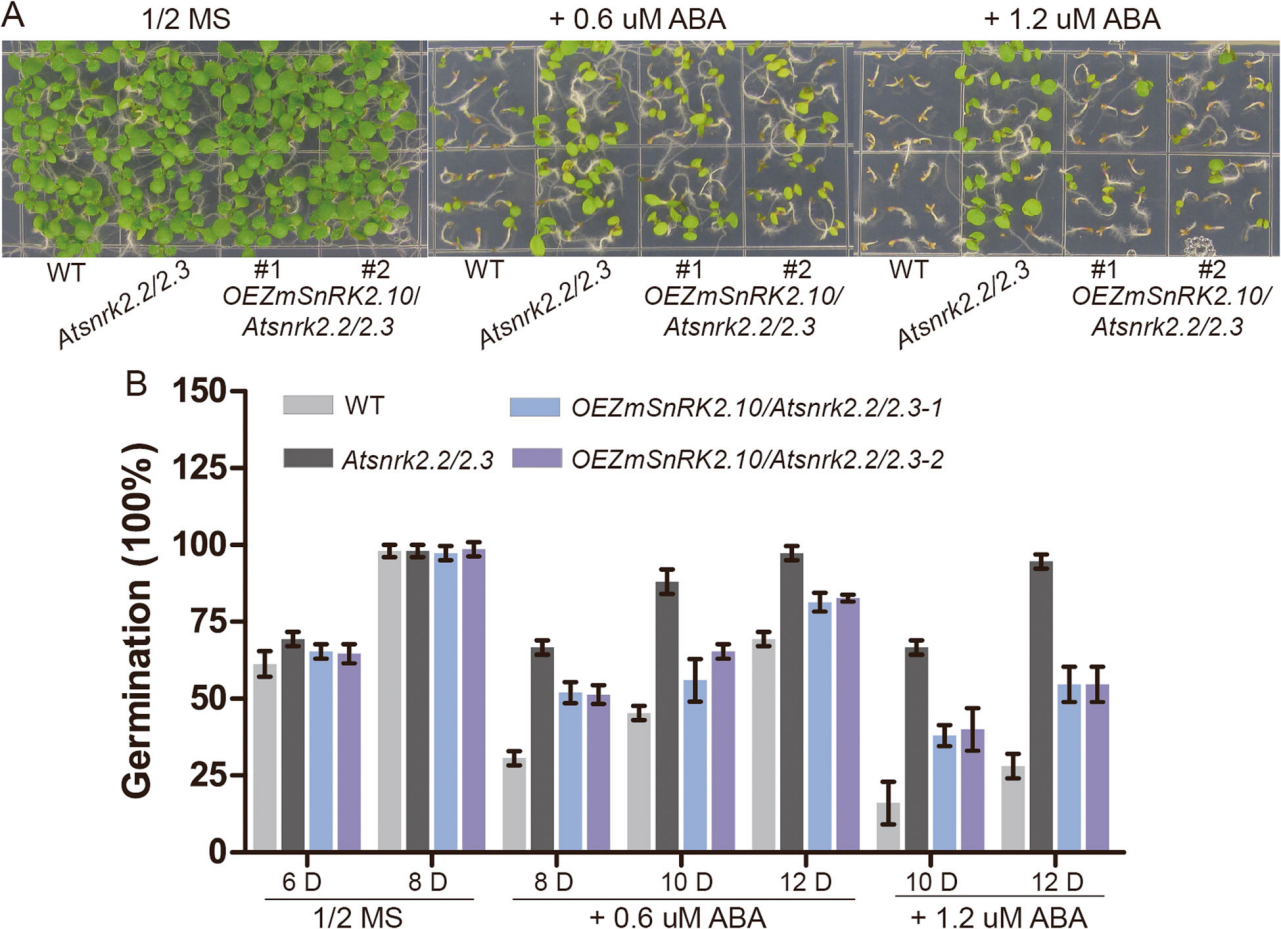
Overexpression of *ZmSnRK2.10* partially rescues the insensitive phenotypes of *Atsnrk2.2/2.3* double mutant to ABA. (**A**) Photographs of the WT, *Atsnrk2.2*/*2.3*, and *OEZmSnRK2.10*/*Atsnrk2.2*/*2.3* seedlings grown on the MS medium (2% sucrose) supplemented without or with ABA (0.6 μM and 1.2 μM). Photographs were taken 7 days after seeds germination. (**B**) The germination rate of WT, *Atsnrk2.2*/*2.3*, and *OEZmSnRK2.10*/*Atsnrk2.2*/*2.3* on the MS medium (2% sucrose) supplemented without or with ABA (0.6 μM and 1.2 μM)

To investigate the biological functions of *ZmSnRK2s*, we generated Arabidopsis transgenic plants expressing four selected *ZmSnRK2* genes (*ZmSnRK2.3/2.8/2.10/2.11*) driven by the Ubi promoter in *snrk2.2/2.3* double mutant background, and several homozygous transgenic lines were obtained for each transgene. The *Atsnrk2.2/2.3* double mutant exhibits ABA-insensitive phenotypes during seed germination and seeding growth [19]. To check whether the overexpression of *ZmSnRK2* genes affects the ABA sensitivity of the *Atsnrk2.2/2.3* double mutant, the seeds from the WT, *Atsnrk2.2/2.3* mutant and transgenic plants were germinated on the media supplemented with different concentrations of ABA. As shown in Additional file 1: Fig. S6, no difference in seeds germination was observed among the WT, *Atsnrk2.2/2.3* mutant and the transgenic plants under normal conditions. In the presence of exogenous ABA, however, the seeds germination and seeding growth of *ZmSnRK2.8* and *ZmSnRK2.10* transgenic plants in *Atsnrk2.2/2.3* mutant background was obviously inhibited compared to *Atsnrk2.2/2.3* mutants, but less serious than the WT. In contrast, the *ZmSnRK2.3* and *ZmSnRK2.11* overexpressing plants in *Atsnrk2.2/2.3* mutant background displayed a similar phenotype as *Atsnrk2.2/2.3* mutant under ABA treatment. These results indicated that *ZmSnRK2.8* and *ZmSnRK2.10* possess a similar function as *AtSnRK2.2* and *AtSnRK2.3* in ABA signaling pathways. The germination rate of *ZmSnRK2.10* overexpression in *Atsnrk2.2/2.3* mutant was further statistically analyzed. As Fig. 6 showed, at the 10th day after sown on 1/2MS agar medium containing 1.2 μM ABA, approximately 50% seeds of the two *OEZmSnRK2.10*/*Atsnrk2.2*/*2.3* lines comparing with 26% of the WT and 100% of *Atsnrk2.2/2.3* mutant seeds germinated, which further confirmed that *ZmSnRK2.10* could partially complement the ABA insensitive phenotype of *Atsnrk2.2/2.3*.

**Fig. 7:**
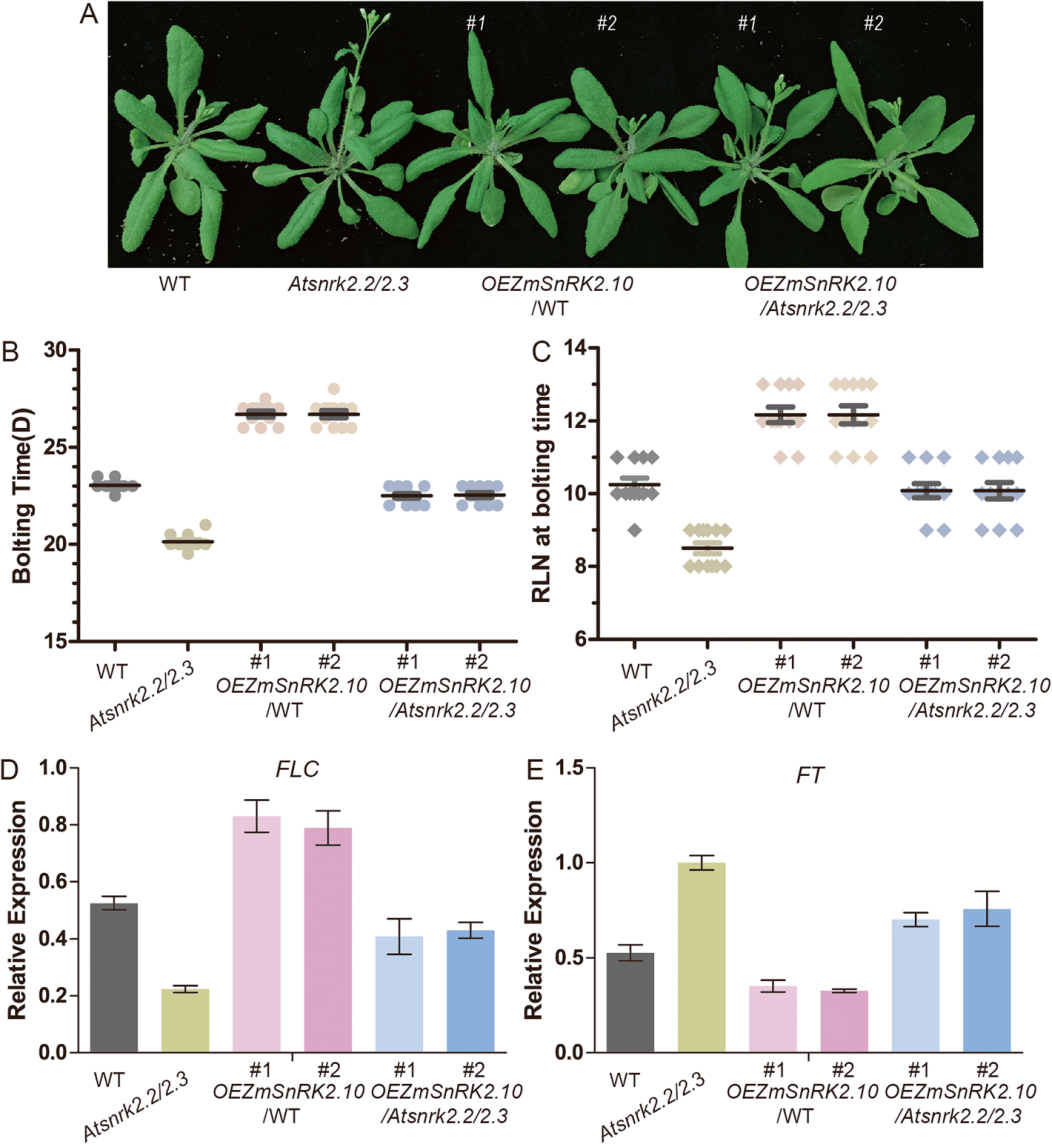
Overexpression of *ZmSnRK2.10* leads to delayed flowering time in Arabidopsis*.* (**A**) Flowering phenotype of the WT, *Atsnrk2.2/2.3*, *OEZmSnRK2.10*/WT, and *OEZmSnRK2.10*/*Atsnrk2.2/2.3*. The photograph was taken 35 days after growth under LD conditions. (**B**) Flowering time of the WT, *Atsnrk2.2/2.3*, *OEZmSnRK2.10*/WT, and *OEZmSnRK2.10*/*Atsnrk2.2/2.3* under LD conditions was scored based on the days from germination to bolting. (**C**) Flowering time of the WT, *Atsnrk2.2/2.3*, *OEZmSnRK2.10*/WT, and *OEZmSnRK2.10*/*Atsnrk2.2/2.3* under LD conditions was scored based on the number of rosette leaves at flowering stage. (**D**) qRT-PCR analysis of *FLC* gene in the WT, *Atsnrk2.2/2.3*, *OEZmSnRK2.10*/WT, and *OEZmSnRK2.10*/*Atsnrk2.2/2.3*. (**E**) qRT-PCR analysis of *FT* gene in the WT, *Atsnrk2.2/2.3*, *OEZmSnRK2.10*/WT, and *OEZmSnRK2.10*/*Atsnrk2.2/2.3*. Error bars represent SE from three biological replicates

We also analyzed the flowering time of the transgenic lines that expressed *ZmSnRK2.10* in the WT and *Atsnrk2.2*/*2.3* mutant background (Fig. 7A-C). Comparing with the WT plants, the *Atnrk2.2*/*2.3* double mutants showed an early flowering phenotype. Overexpression of *ZmSnRK2.10* in *Atsnrk2.2*/*2.3* background, however, could restore the flowering time of the *Atsnrk2.2*/*2.3* double mutants to WT level. *ZmSnRK2.10* in WT background also resulted in a significant delay of flowering time. In the transgenic plants overexpressing *ZmSnRK2.11* gene in both WT and *Atsnrk2.2*/*2.3* mutant background, the flowering time was not affected. (Additional file 1: Fig. S7), suggesting that *ZmSnRK2.10* has a special role in controlling flowering time in Arabidopsis. To understand the mechanism of *ZmSnRK2.10* in the regulation of flowering time, the transcript levels of two critical genes, *FLOWERING LOCUS T* (FT) and *FLOWERING LOCUS C* (*FLC*), in transgenic lines were analyzed by qRT-PCR. *FT* controls flowering time in Arabidopsis, and its mutant plants show significantly late flowering [33]. *FLC* is a floral repressor and *FT* can directly bind to the promoter region of *FLC* to inhibit its expression, thus inhibiting the flowering in Arabidopsis [34]. The qRT-PCR analysis indicated that the expression level of *FLC* was up-regulated and the expression level of *FT* was reduced in the transgenic plants overexpressing *ZmSnRK2.10* (Fig. 7D-E). Therefore, we suspected that overexpression of *ZmSnRK2.10* delayed flowering time at least partially via the regulation of the expression of *FLC* and *FT* genes.

**Fig. 8:**
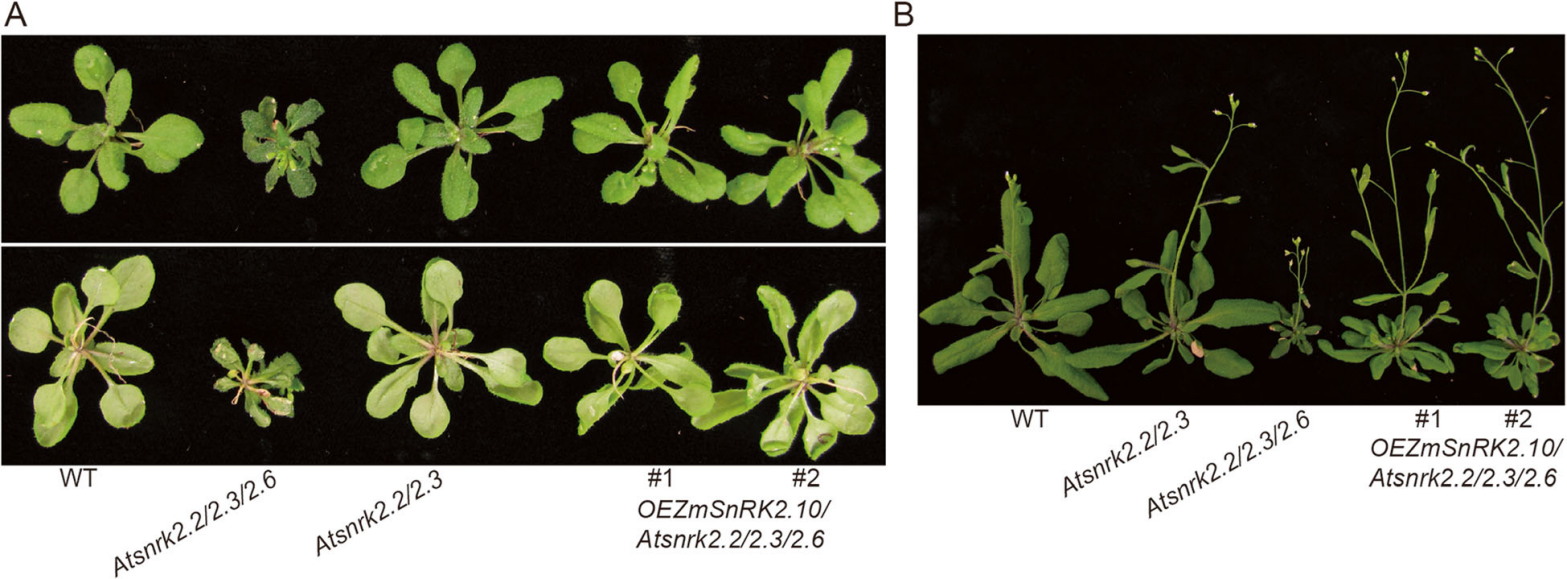
Overexpression of *ZmSnRK2.10* partially rescues the phenotype of *Atsnrk2.2/2.3/2.6* in Arabidopsis. (**A**) The phenotype of the WT, *Atsnrk2.2/2.3, Atsnrk2.2/2.3/2.6,* and *OEZmSnRK2.10*/*Atsnrk2.2/2.3/2.6*. The photograph was taken 30 days after growth under LD conditions. (**B**) Flowering phenotype of the WT, *Atsnrk2.2/2.3*, *Atsnrk2.2/2.3/2.6*, and *OEZmSnRK2.10*/*Atsnrk2.2/2.3/2.6*. The photograph was taken 35 days after growth under LD condition

In addition, nearly all major ABA responses are blocked in the *Atsnrk2.2/2.3/2.6* triple mutant in Arabidopsis. The triple mutant shows earlier flowering, smaller plant size, fewer pollen number, and poor fertilization phenotype compared with *Atsnrk2.2*/*2.3* double mutant [6, 16]. We also transformed *ZmSnRK2.10* into *Atsnrk2.2*/*3*/*6* triple mutant and obtained overexpression transgenic lines (Additional file 1: Fig. S8A, B). Overexpression of *ZmSnRK2.10* rescued the dwarf morphology of *Atsnrk2.2/2.3/2.6* mutant significantly, but the plant size of transgenic plants was still smaller than the WT (Fig. 8 and Additional file 1: Fig. S8C). This result indicated that *ZmSnRK2.10* functions redundantly with *AtSnRK2.2/2.3/2.6.*

## Discussion

### The conserved evolution of the *ZmSnRK2s*

The core components of ABA signaling pathway have been well characterized in the dicot model plant Arabidopsis, but the studies on ABA regulatory pathway in crops still need to be strengthened. Protein kinase SnRK2s is the central node of ABA signal transduction [35, 36]. The *SnRK2* genes family has been identified in many plants. In maize, 11 *SnRK2* genes have been reported previously [26]. In this study, we identified 14 members of *SnRK2* at genome-wide level using the latest maize genome database. Compared with 10 members in Arabidopsis and 10 members in rice, the *SnRK2* members in maize were expanded to 14 [9, 10]. The expansion of *SnRK2* genes in maize was attributed to gene duplication events, which is one of the important strategies that plants used to adapt to the surrounding environment [37, 38]. It has been reported that three duplication events, including whole-genome duplication, genomic tandem duplication and segmental duplication in plants [39]. Similar to Arabidopsis and rice, the *SnRK2* genes in maize were divided into three subclasses (Additional file 1: Fig. S1 and Fig. S2), and the exon-intron organizations of *SnRK2* genes among these three species exhibited a high similarity (Fig. 2A), which indicates that the gene structures of *SnRK2s* are evolutionarily conserved in higher plant. Further, the collinearity relationship of *SnRK2s* indicated that *SnRK2* genes had been differentiated before the differentiation of maize, rice and Arabidopsis (Fig. 2C). The most ancient land plants, such as *Bryophytes* and *Charophyceae algae*, only contain *SnRK2* genes clustered in subclass III, and the subclasses I and II branches were evolved as new systems to adapt to osmotic stress conditions after the separation of bryophytes and vascular plants [38]. The neofunctionalization and subfunctionalization of the duplication gene is an important mechanism to maintain its stability. The original duplicated gene has dual functions and repulsion between these two functions, which results in gene loss. When the duplication event occurs, each gene obtains one of the functions and optimizes them respectively, leading to the elimination of functional conflict and the stability of duplicated genes [40]. This indicates that the duplication events of *SnRK2* genes represent an ancient system and have expanded along with whole-genome duplication events in plants. Previous studies speculated that maize had experienced at least three genome-wide replication events: monocotyledonous plant differentiation, the emergence of *Gramineae*, and the differentiation of maize and sorghum. These duplication events occurred about 110 million years ago, 50 million years ago, and 12 million years ago, respectively [41–43]. In addition, through the synteny and collinearity analysis of *SnRK2* genes in maize genome, we identified eight segmental duplications but no tandem duplication (Fig. 2B), which supports the opinion that most of maize genes were duplicated during ancient polyploidization [44]. These results suggested that the expansion of *SnRK2* members and origin of novel gene functions in maize maybe due to the segment duplication and polyploidization. This explanation is consistent with the adaptive radiation model, which proposes that the newly developed genes help the existing genes to cope with new conditions, and the genomic tandem duplication accelerates the evolution of novel function of the duplicated genes [45, 46].

### The expression pattern of *ZmSnRK2s* in maize

Analysis of gene expression patterns can provide important clues to explore gene function. In Arabidopsis, the tissue-specific expression of *SnRK2.6* in stomata is tightly coupled with its functions in guard cells to regulate stomatal closure [18]. The expression of two other subclass III genes, *SnRK2.2* and *SnRK2.3*, are different from that of *SnRK2.6*. These two genes express in nearly all the tissues and are closely related to their role in regulating various ABA responses, such as seed germination, dormancy, and seedling growth [19, 28]. Previous studies revealed the expression profile of *ZmSnRK2* family genes under different abiotic stresses [26, 31]. To investigate the spatiotemporal expression patterns of *ZmSnRK2s*, we first analyzed the expression of *ZmSnRK2s* using public RNA-Seq database, and found that *ZmSnRK2* genes exhibited differential expression patterns during the growth and development of various maize tissues. Even in the same subclass, each gene exhibited a distinct expression pattern. The paralogous genes tend to maintain similar functions, but show differences in activity and specificity. The significant difference in gene expression among *ZmSnRK2* genes is consistent with the results reported in previous studies [47, 48]. The changes of homologous genes in the promoter region lead to rapid divergence in expression patterns, which further lead to functional differentiation [49]. The chromosomal location, gene structure, and collinearity analysis indicated that four genes *ZmSnRK2.8*, *ZmSnRK2.9*, *ZmSnRK2.10*, and *ZmSnRK2.12,* which belong to subclass III of SnRK2 in maize, are derived from gene duplication events. We focused on the differential expression pattern of these four genes and found that *ZmSnRK2.10* was highly expressed in both embryo and endosperm, especially in the middle and late stage of kernel development, the stages of which are important for storage material accumulation and seed maturation (Fig. 3D). In addition, the transcripts of *ZmSnRK2.10* were induced by ABA and sucrose respectively (Additional file 1: Fig. S3). In wheat, the transcripts of *PKABA1* and *TaSnRK2.3* were induced by ABA, and both genes are important for seed maturation and abiotic stress response [8, 27]. Although the different maize tissues were tested in this research and previous reports [26, 31], the expression pattern of some *ZmSnRK2s* under ABA treatment is almost consistent, such as *ZmSnRK2.1* and *ZmSnRK2.7* (Additional file 1: Fig. S3). Moreover, the expression pattern of most *ZmSnRK2s* in maize different tissues varied. For instance, the *ZmSnRK2.10* showed an increased expression in kernel after ABA treatment, but was downregulated expression in seedling, leaf and root [26, 31]. Combined with the results that *ZmSnRK2.10* was highly expressed in kernel and was induced by ABA, we propose that *ZmSnRK2.10* plays important roles in linking metabolism and stress signaling during the grain filling and maturation process.

### Functional characterization of *ZmSnRK2.10* in Arabidopsis

*SnRK2s* are divided into three subclasses based on ABA activation in Arabidopsis*.* Subclass III, including *SnRK2.2*, *SnRK2.3* and *SnRK2.6*, are strongly activated by ABA, and subclass II, including *SnRK2.7* and *SnRK2.8*, are slightly activated by ABA. The subclass I *SnRK2s* cannot be activated by ABA, but can be strongly activated by hyperosmotic stress except the *SnRK2.9* [7, 11]. Because the subclass III SnRK2s are ABA-dependent, the *snrk2.2* or *2.3* single mutant exhibits weak ABA-insensitive phenotypes and *snrk2.2/2.3* double mutant shows strong ABA-insensitive phenotypes during seed germination and seedling growth in Arabidopsis [19]. Further studies showed that *snrk2.2/2.3/2.6* triple mutant exhibits almost completely insensitive to ABA in seed germination and seedling growth [16, 28]. *SAPK8*, *SAPK9*, and *SAPK10*, which are homologs of *AtSnRK2.2, AtSnRK2.3*, and *AtSnRK2.6*, belong to subclass III in rice and are strongly activated by ABA [10]. Overexpression of *SAPK8*, *SAPK9*, or *SAPK10* in rice leads to delayed germination and seedling growth, which are caused by the accumulation of higher levels of ABA compared with WT plants [21]. Compared with *SAPK8* and *SAPK9*, *SAPK10* displays the highest homology to *AtSnRK2.2* and *AtSnRK2.3* and it mediates the phosphorylation of WRKY72, which results in the release of its suppression on jasmonic acid biosynthesis and bacterial blight resistance [50]. Overexpression of *SAPK10* confers rice with hypersensitivity to ABA during seed germination and seedling growth [51]. In maize, we found that the *ZmSnRK2.10* was the closest homologous gene of *SAPK10* in rice and *SnRK2.2/2.3* in Arabidopsis*,* and this gene, as well as *ZmSnRK2.8* (same as *OST1* in Arabidopsis), was activated by ABA. The other subclass members *ZmSnRK2.3* and *ZmSnRK2.11* cannot be activated by ABA (Fig. 5 and Additional file 1: Fig. S5). Overexpression of *ZmSnRK2.8* rescues the drought-hypersensitive phenotype of *ost1* mutant and significantly improves the growth and development of plants under stress conditions in Arabidopsis [32, 52] In our study, overexpression of *ZmSnRK2.8* and *ZmSnRK2.10*, but not *ZmSnRK2.3* and *ZmSnRK2.11*, in *AtSnRK2.2/2.3* background can partially complement the hypersensitive phenotypes of *Atsnrk2.2/2.3* to ABA in seed germination and early seedling development (Fig. 6 and Additional file 1: Fig. S6). In addition, overexpression of *ZmSnRK2.10* can also rescue the phenotype of *Atsnrk2.2/2.3/2.6* triple mutant (Fig. 8 and Additional file 1: Fig. S8). The identified functions of *ZmSnRK.10* in Arabidopsis suggests that the ABA-dependent members of *SnRK2* maybe are evolutionarily conserved in plants.

On the other hand, it has been reported that ABA can inhibit plant floral transition [53]. Exogenous application of ABA delays plant flowering time. A series of ABA synthesis and signal transduction mutants, including *aba2*, *abi4*, *abi5*, and *snrk2.2/2.3/2.6*, exhibit early-flowering phenotype [6, 53–55]. We found that the *snrk2.2/2.3* double mutant also showed early flowering in Arabidopsis and overexpression of *ZmSnRK2.10* in *snrk2.2/2.3* double mutant rescued early-flowering phenotype. Moreover, overexpression of *ZmSnRK2.10* in WT background delayed flowering time via the upregulation of *FLC* gene that is involved in floral transition (Fig. 7). This result is consistent with the interpretation that SnRK2-mediated phosphorylation of ABI5 and/or ABF promotes *FLC* gene expression [53]. All these results indicate that molecular functions of subclass III SnRK2s are conserved in plants. In future, overexpression or knock-out of *ZmSnRK2.10* by CRISPR-Cas9 technique needs to be performed in maize, which will advance our understanding of the roles of *ZmSnRK2.10* in the regulation of abiotic stress tolerance in maize.

## Conclusion

Although *ZmSnRK2s* have been cloned and characterized in maize as early as 2008, no genome-wide identification and characterization of *ZmSnRK2s* has been conducted in maize. Here, we presented a genome-wide identification of *ZmSnRK2s* in maize and found 14 *SnRK2* genes based on the released maize genome data. Through chromosomal localization, gene structure, evolutionary association, synteny and collinearity analysis of *SnRK2* genes in maize by comparing with the *SnRK2s* in rice and Arabidopsis, we can learn that the *SnRK2* family genes are evolutionarily conserved in plants, especially the ABA-dependent subclass III SnRK2s. In addition, through transcriptional and biochemical analysis, we found that *ZmSnRK2.10* was highly expressed in kernel and was activated by ABA. Overexpression of *ZmSnRK2.10* in *snrk2.2/2.3* double and *snrk2.2/2.3/2.6* triple mutants partially rescued the ABA-insensitive phenotypes of these mutants, and also resulted in delaying plant flowering in Arabidopsis. Our study provided new insights about the structure, evolution, and functions of *SnRK2s* in maize.

## Methods

### Plant materials and growth condition

Maize (*Zea mays* L.) self-interbreeding line B73 material (obtained from Maize Research Institute of Sichuan Agricultural University) used in this study, was grown on the experimental farm of Sichuan Agricultural University in Chongzhou, Sichuan, China. Seeds of *Arabidopsis thaliana* accession Col-0 (Columbia 0) and the mutants *snrk2.2/2.3*, *snrk2.2/2.3/2.6* (all in Col-0 background, obtained from Zhu lab in Purdue University) [6, 19] were used in this study. The *Arabidopsis* plants, including transgenic lines, were grown under 16 h light/8 h dark condition at 22 °C in the day and 16 °C at night.

### Whole genome identification of SnRK2s in *Zea mays*

To identify the *SnRK2* genes in *Zea mays*, we obtained the genome sequence and annotation data of *Zea mays* from MaizeGDB (https://www.maizegdb.org/). The protein database and genome database were blasted by using the query sequences of the *SnRK2* family in *Arabidopsis thaliana* (http://www.arabidopsis.org) and *Oryza sativa* (http://www.ricedate.com). The E-value cut-off was set at 1.0e-5 to ensure confidence and redundant sequences were deleted by manual.

### Multiple sequence alignment, orthologous gene identification and phylogenetic analysis

The phylogenetic analysis of SnRK2 family were conducted as the method in this article [56]. In brief, amino sequences from *Zea mays*, *Arabidopsis thaliana* and *Oryza sativa* were aligned by ClustalX v1.83 with default parameters and then constructed a phylogenetic tree using the neighbor joining (NJ) method in MEGA (version 5.10) [57]. The parameters of NJ analysis were as followed: bootstrap with 1000 replicates for statistical testing, pairwise deletion for data processing and poisson correction for modeling. The position in phylogenetic tree (bootstrap value > 50) and identify between orthologous gene pairs (> 90%) would determine the orthologous gene in *Zea mays*, *Arabidopsis thaliana* and *Oryza sativa*.

### Chromosomal location of ZmSnRK2s and collinearity analysis

Gene ID information of the *ZmSnRK2* loci on chromosome were obtained from the genome annotation files. The distribution of *ZmSnRK2s* on the chromosome was generated with MapChart version 2.32 [58]. The Gene Structure Display Server (http://gsds.cbi.pku.edu.cn/) was used for gene structure analysis. Conserved protein motifs of the *ZmSnRK2s* were predicted by the MEME Version 5.0.4 program (http://meme-suite.org/). The default parameters of MEME were setting except two: maximum number of motifs, 25; optimum width, 6–50 [59].. The MCscanX program was used to identify *ZmSnRK2s* duplications as previous described [56, 60]. All genes were classified into various types of duplications, including WGD, segmental and tandem duplications. A schematic diagram of the putative WGD or segmental duplications of *ZmSnRK2s* was constructed using the Circos (http://circos.ca/), and the *ZmSnRK2s* with WGD or segmental duplications were linked by lines.

### Analysis of the expression pattern of *ZmSnRK2* genes

To study the expression patterns of the *ZmSnRK2* genes, we obtained the expression level of each *ZmSnRK2* gene in different tissues by analyzing the released transcription data [29], and then submitted it to the MeV_4_9_0 [61] for stratified clustering (Hierarchical Clustering, HCL). Further, different tissues of B73 were collected for RNA extract and qRT-PCR analysis, including root, stem, leaf, filament, anther at the same stage (Heading stage). Besides, the development kernels were also collected, including kernels at 2–30 days after pollination (DAP), endosperm and embryo at 15 DAP. The treatment to vitro kernel was according to the paper described by [30]. The RNA extraction of materials was followed the protocol of Trizol (TIANGEN, Beijing, China) reagent. RNA was reverse transcribed using the PrimeScript™ RT Reagent Kit with genomic DNA Eraser (Takara, Dalian, China), and quantitative RT-PCRs were performed using a SYBR premix Ex Taqtm RT-PCR kit (Takara, Dalian, China). All the experiments were performed following the manufacturer’s instructions. The primers used for qRT-PCR were listed in Additional file 1: Table S2.

### Gene cloning, vector construction and subcellular localization of ZmSnRK2 genes

Full-length cDNA sequence of *ZmSnRK2* genes exclude terminator codon were amplified by PCR. The PCR products were sequenced and then sub-cloned into the pCAMBIA 2300-35S-eGFP vector [62]. Then the pCAMBIA2300-ZmSnRK2-eGFP fusion protein were transformed into *Agrobacterium tumefaciens* strain GV3101. The empty pCAMBIA2300-35S-eGFP was used as a control. The *Agrobacterium* was infected into *Nicotiana benthamiana* leaves, and the fluorescence signal of green fluorescent protein (GFP) was observed by confocal microscope (Leica, Wetzlar, Germany). The *ZmSnRK2.14* was not subcloned due to its low expression in almost all tissues. The primers used for vector contractor were listed in Additional file 1: Table S2.

### Isolation and transfection of protoplast

The isolation of protoplasts in maize were performed as described with minor modification [63]. The middle parts (7 cm) of the second leaves of B73 were transversally cut into 0.5–0.8 mm long strips and put into a petri dish containing 0.6 M mannitol for 5 min. Then the strips were transferred into another dish containing enzyme solution [0.6 M mannitol, 20 mM MES, pH 5.7, 0.1% cellulase RS, 0.01% macerozyme, 0.1% BSA, 1 mM CaC1_2_ and ddH_2_O]. After vacuum (15–20 cm-Hg) distillation at room temperature for 20 min, the dish was incubated at 28 °C in the dark with shaking for 3 h at 40 rpm and for 10 min at 80 rpm. Then, the protoplasts were released. The enzyme solution containing protoplasts was transferred into sterile tubes by filtering with 35 μm nylon mesh. The protoplasts were collected by centrifugation at 110×g for 4 min at room temperature and washed once with 0.6 M mannitol. Finally, the protoplasts were resuspended in MMg solution (0.6 M mannitol, 4 mM MES, pH 5.7, 15 mM MgC1_2_) and observed under a light microscope to detect the quality and quantity.

The transfection of protoplast was performed with PEG-mediated protoplasts transformation protocol [63, 64]. The 20 μg plasmid DNA of p2300-ZmSnRK2s were mixed with 200 μL protoplasts (a concentration of 2× 10^5^ cells/mL). About 220 μL PEG solution (0.8 M mannitol, 40% PEG 4000 and 100 mM CaCl_2_,] was immediately mixed with the protoplasts and plasmid DNA by gently shaking, and then incubated for 15 min at 25 °C. After incubation, the mixture of protoplasts was washed and collected by centrifugation, and then resuspended in incubation solution (4 mM MES, pH 5.7, 0.6 M mannitol, 4 mM KCl) and incubated in the dark at 25 °C overnight. The fluorescence signal was observed by the confocal microscope (Leica, Wetzlar, Germany).

Co-transfected ZmSnRK2.3, ZmSnRK2.8, ZmSnRK2.10 and ZmSnRK2.11 with reporter gene to reconstitute the ABA signaling pathway in B73 leaves protoplast respectively. The ABA-responsive reporter and internal control were selected with RD29B-LUC and ZmUbi-GUS respectively. After transfection, the protoplasts were incubated in the absence/ presence of 50 μM ABA for 2 h under light for further analysis.

### Construction of transgenic plants

The sequencing-confirmed ORFs of *ZmSnRK2.3*, *ZmSnRK2.8*, *ZmSnRK2.10* and *ZmSnRK2.11* were cloned into the pCAMBIA3301 vector (CAMBIA, Canberra, Australia) respectively. The constructs were transferred into *Agrobacterium tumefaciens* strain GV3101 using electroporation method and then transformed into *Arabidopsis* using the floral tip method [65]. The seeds of the T_0_ generation were harvested and sown in soil, and the transformants T_1_ plants were identified by spraying with Basta (glufosinate ammonium; Solarbio, Beijing, China) and confirmed by PCR to amplify *35S:ZmSnRK2s* fragment and bar gene, respectively. The confirmed transgenic plants were harvested individually. The T_2_ seeds were placed on 1/2MS (Murashige & Skoog) agar medium (1%) containing 5 mg/L glufosinate ammonium. Seeds of T_3_ were collected from transgenic lines with a 3:1 (resistant: sensitive) segregation ratio. The two individual T_3_ lines displaying 100% Basta resistance were considered homozygous and used for further experiments. The *Arabidopsis* Col-0 was used as control. All seeds of WT, mutants and transgenic plants were collected at the same stage.

### Germination assays and phenotype identification

Seeds were sterilized for 20 min with 75% (v/v) ethanol and 0.1% (v/v) Tween 80, then washed with ethanol twice and air-dry. Fifty seeds of each transgenic line, mutant and WT were placed on the 1/2MS agar medium (1.0%) supplemented with different concentrations of ABA (0.5, 0.6, 1.0, 1.2 or 1.5 μM), maintained at 4 °C in dark for 2 days, and then transferred to the growth chamber at 65% relative humidity under 16-h -light at 22 °C and 8-h-dark at 18 °C. The percentage of emergence of two green cotyledons was measured daily at 8 days after seeds were placed at room temperature.

The flowering time phenotype of WT, mutants and transgenic lines were compared by measuring the bolting time of each lines. Recording the numbers of leaf (RLN) would be another trait to measure the flowering time.

### GUS-staining assays

In the GUS assay, GUS activity was performed following this paper [66] with few modifications. The cutting leaves were transferred into a GUS staining solution (1 mM X-Gluc, 10 mM phosphate buffer [pH 7.0], 0.5% [v/v] Triton X-100 and 2 mM potassium ferricyanide). After vacuum infiltration and overnight shaking incubation at room temperature, the leaves were de-stained by repeated washes in 70% ethanol and photographed.

### Protein extraction and immunoblot analysis

For the experiment shown in Fig.5, Additional Files: Fig. S6, S7 and S8, protein extraction and immunoblot analysis were performed as described by [11] with minor modifications. The protein extraction buffer (100 mm HEPES, pH 7.5, 5 mm EDTA, 5 mm EGTA, 2 mm orthovanadate, 10 mm NaF, 20 mm β-glycerophosphate, 5 mm DTT, protease inhibitor cocktail [Sigma-Aldrich] for plant cell and tissue extracts 1:100, and 50 μM PMSF) was used to extracted the liquid nitrogen grounded leaves of transgenic plants. Samples were centrifuged at 4 °C and high speed for 10 min, and supernatant was collected. Protein concentration was determined using Bio-Rad protein assay. Immediately after isolation, about 15 μg protein fractions were transferred to SDS loading buffer and separated by SDS-PAGE (12.5% acrylamide). The western blotting detection protein was performed referring to this paper [30].

## Supplementary Information

**Additional file 1.**

## Data Availability

The datasets supporting the conclusions of this study are available by contacting with the corresponding author (yubihuang@sohu.com). The RNA-Seq data are available in NCBI (www.ncbi.nlm.nih.gov) with the accession number SRP037559.

## Abbreviations

ABA: abscisic acid
BSA: bovine serum albumin
CaMV: cauliflower mosaic virus
CDS: sequence coding for amino acids in protein
DAPI: 4′, 6-diamidino-2-phenylindole
EDTA: ethylene diamine tetraacetic acid
EGTA: ethylene glycol-bis(2-aminoethylether)-N, N, N′, N′-tetraacetic acid
GUS: β-glucuronidase
HEPES: 2-[4-(2-hydroxyethyl) piperazin-1-yl] ethanesulfonic acid
MES: morpholinoethanesulfonic acid
MS: Murashige & Skoog
ORF: open reading frame
PEG: polyethylene glycol
PMSF: Phenylmethanesulfonyl fluoride
SDS-PAGE: sodium dodecyl sulfate polyacrylamide gel electrophoresis
Ubi: ubiquitin
