# Supplementary material for: Genome-wide identification of ZmSnRK2 genes and functional analysis of ZmSnRK2.10 in ABA signaling pathway in maize (Zea mays L)
Source: BMC Plant Biol. 2021 Jul 1;21:309. doi: 10.1186/s12870-021-03064-9 (PMC8246669; doi:10.1186/s12870-021-03064-9)
Supplement: Supplementary file 1 — Additional file 1. [file 12870_2021_3064_MOESM1_ESM.docx]

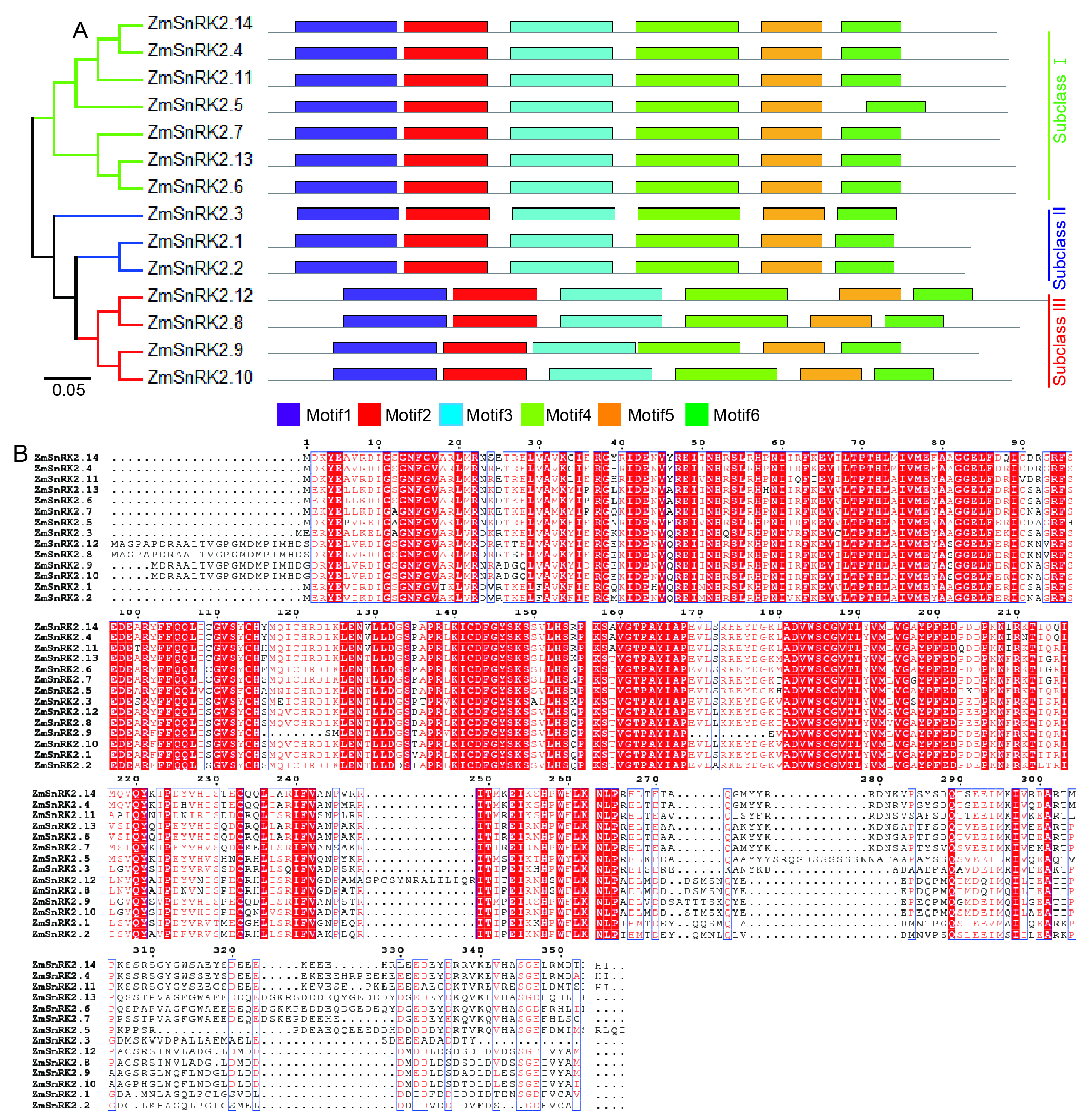


Fig. S1 Phylogenetic tree, conserved motifs and amino acid sequence alignment of SnRK2s in Maize. (A) Phylogenetic tree and analysis the conserved motifs of SnRK2s in maize. Subclass I SnRK2s are colored with green, Subclass II SnRK2s are colored with blue, and Subclass III SnRK2s are colored with red. (B) Amino acid sequence alignment of SnRK2s family in maize.


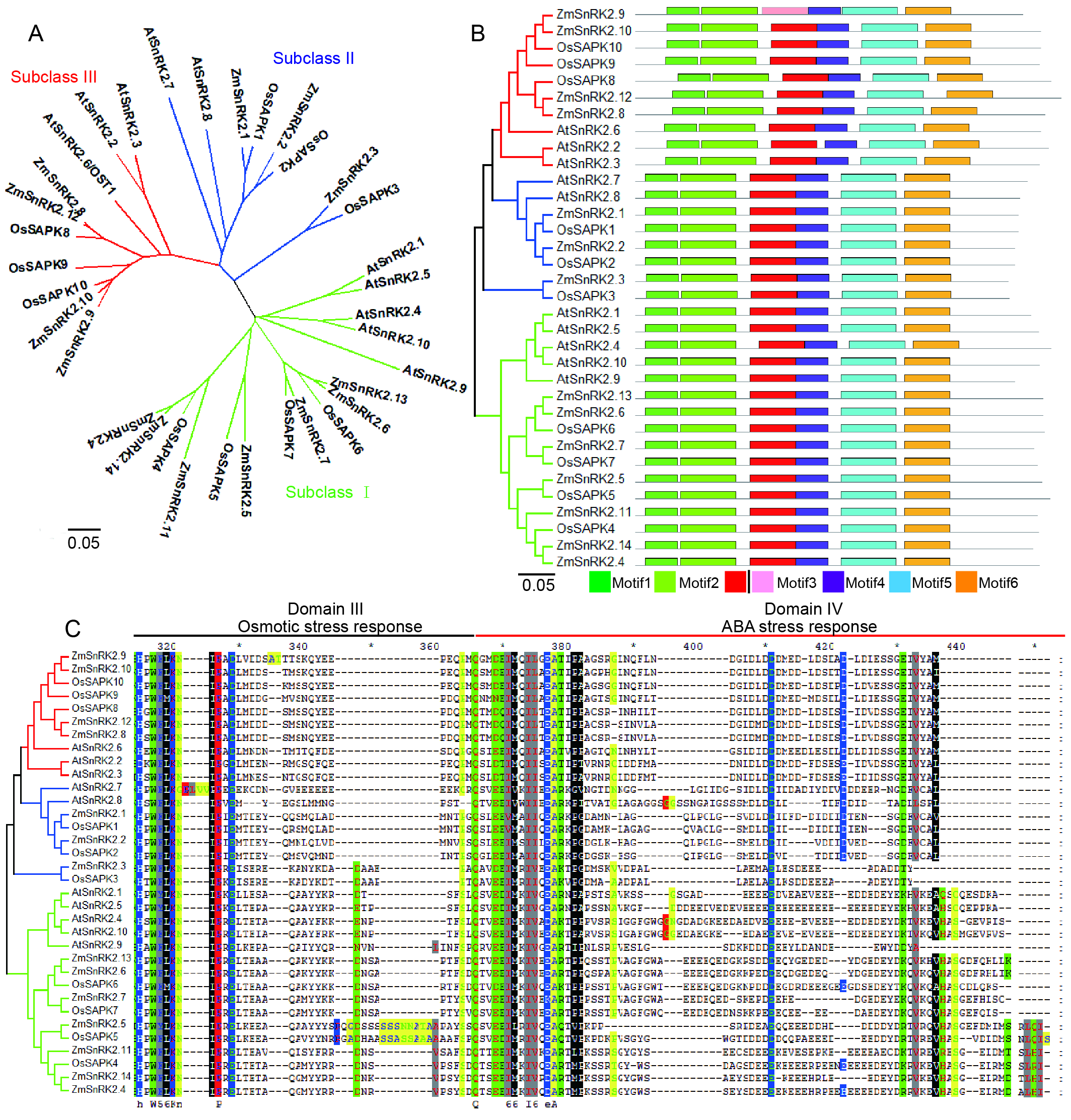


Fig. S2 Phylogenetic relationships, conserved motifs, and amino acid sequence alignment of SnRK2s in maize, rice and *Arabidopsis*. (A) Phylogenetic tree of SnRK2s family in maize, rice and *Arabidopsis*. (B) Conserved motifs analysis of SnRK2 proteins in maize, rice and *Arabidopsis*. (C) Amino acid sequence alignment of the C-terminal domain of SnRK2 proteins. Domain III is responsible for osmotic stress response, and domain IV functions in ABA stress response.


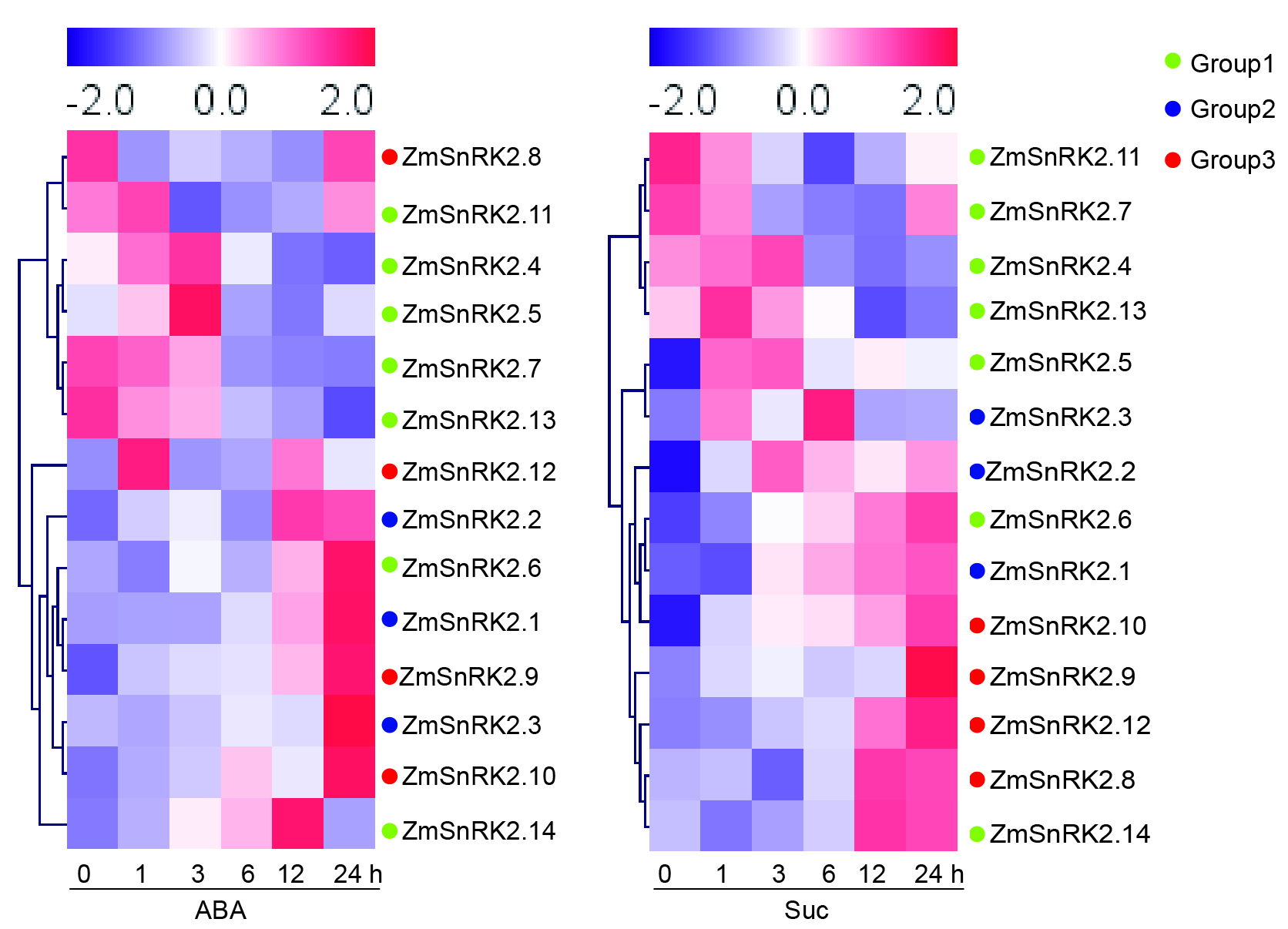


Fig. S3 Analysis of the expression patterns of *ZmSnRK2* genes in maize kernel at different time after ABA or sucrose treatment. These data were derived from three biological replicates.


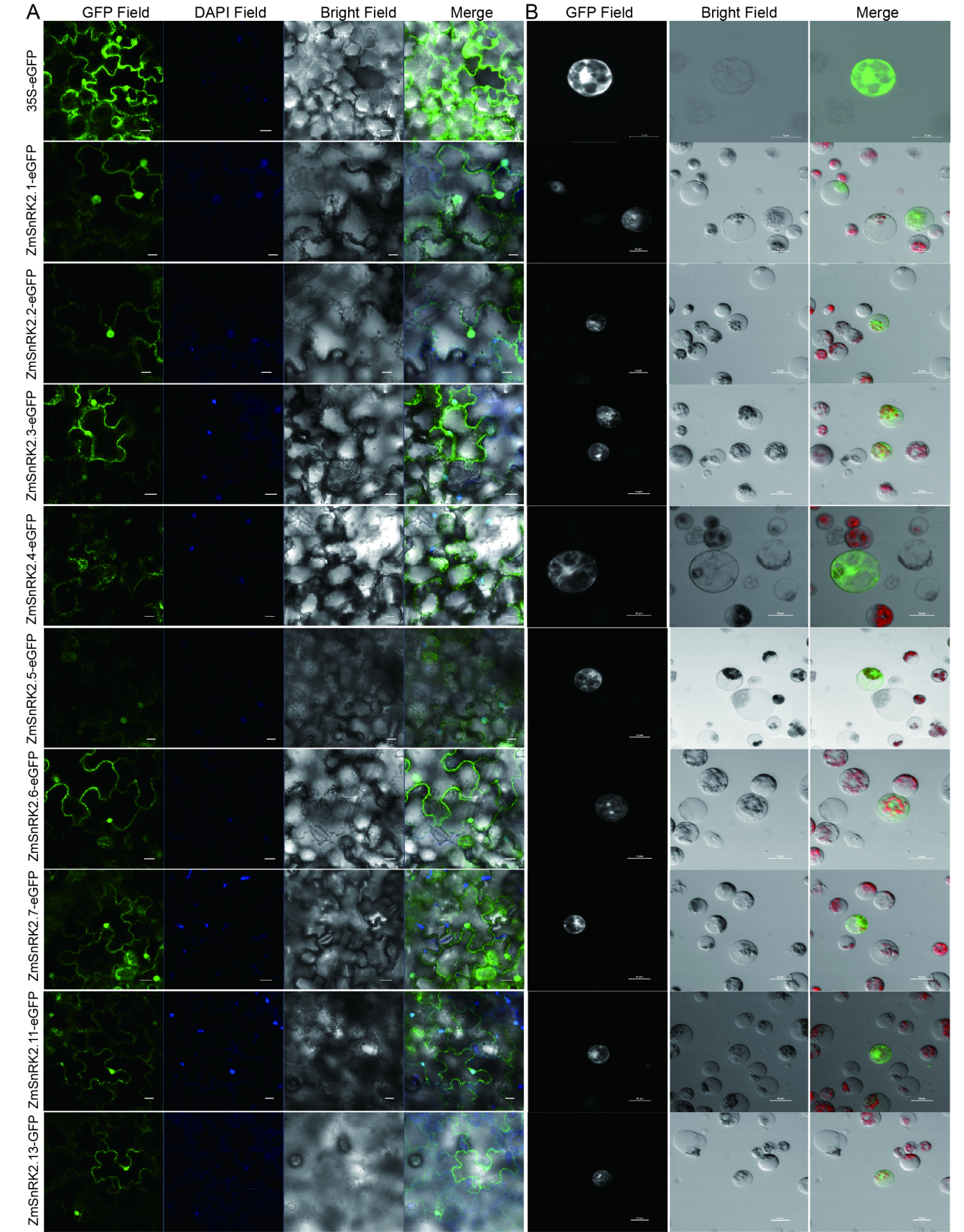


Fig. S4 Subcellular localization of subclass Ⅰ and subclass II ZmSnRK2 proteins. (A) The construct containing ZmSnRK2s:GFP fusion protein vector into the *Agrobacterium tumefaciens* strain GV3101 and used to infect *Nicotiana benthamiana* leaf cell. The bars, 50 µm. (B) The ZmSnRK2s:GFP fusion protein was transformed into maize leaf protoplasts respectively. The bars, 10 µm. All fluorescence signals were detected using the Confocal microscope. *35S-eGFP* was used as the control. Merged, individual images of GFP and bright field images of onion epidermal cells were shown. GFP, green fluorescent protein. The localization of the nuclei was indicated by DAPI (6-diamidino-2-phenylindole) staining.


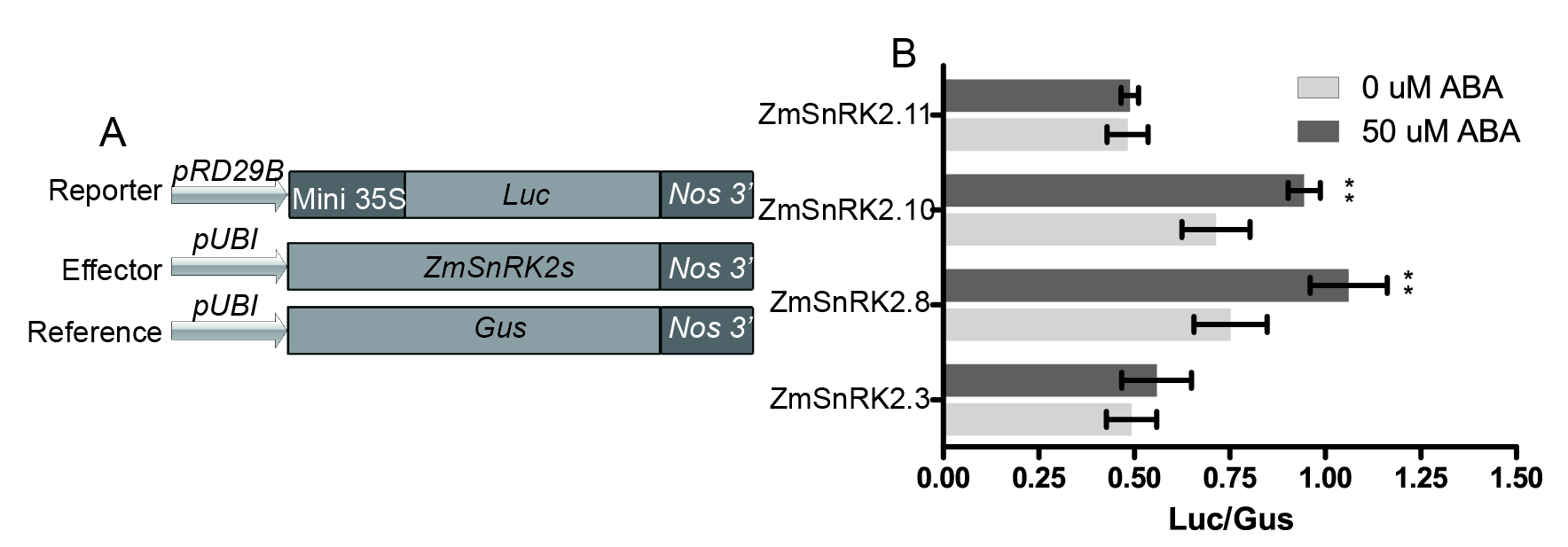


Fig. S5 *ZmSnRK2.10* mediates ABA gene expression. (A) Schematic illustration of the constructs. (B) Activation of the RD29B-Luc reporter gene by *ZmSnRK2s* in the protoplast of maize. The reporter gene expression was examined in maize leaf protoplasts after co-transformation with *ZmSnRK2.3*, *ZmSnRK2.8*, *ZmSnRK2.10*, or *ZmSnRK2.11*. Error bars indicate SEM (n=3). The significant difference was analyzed by t test (*P < 0.05; **P < 0.01).


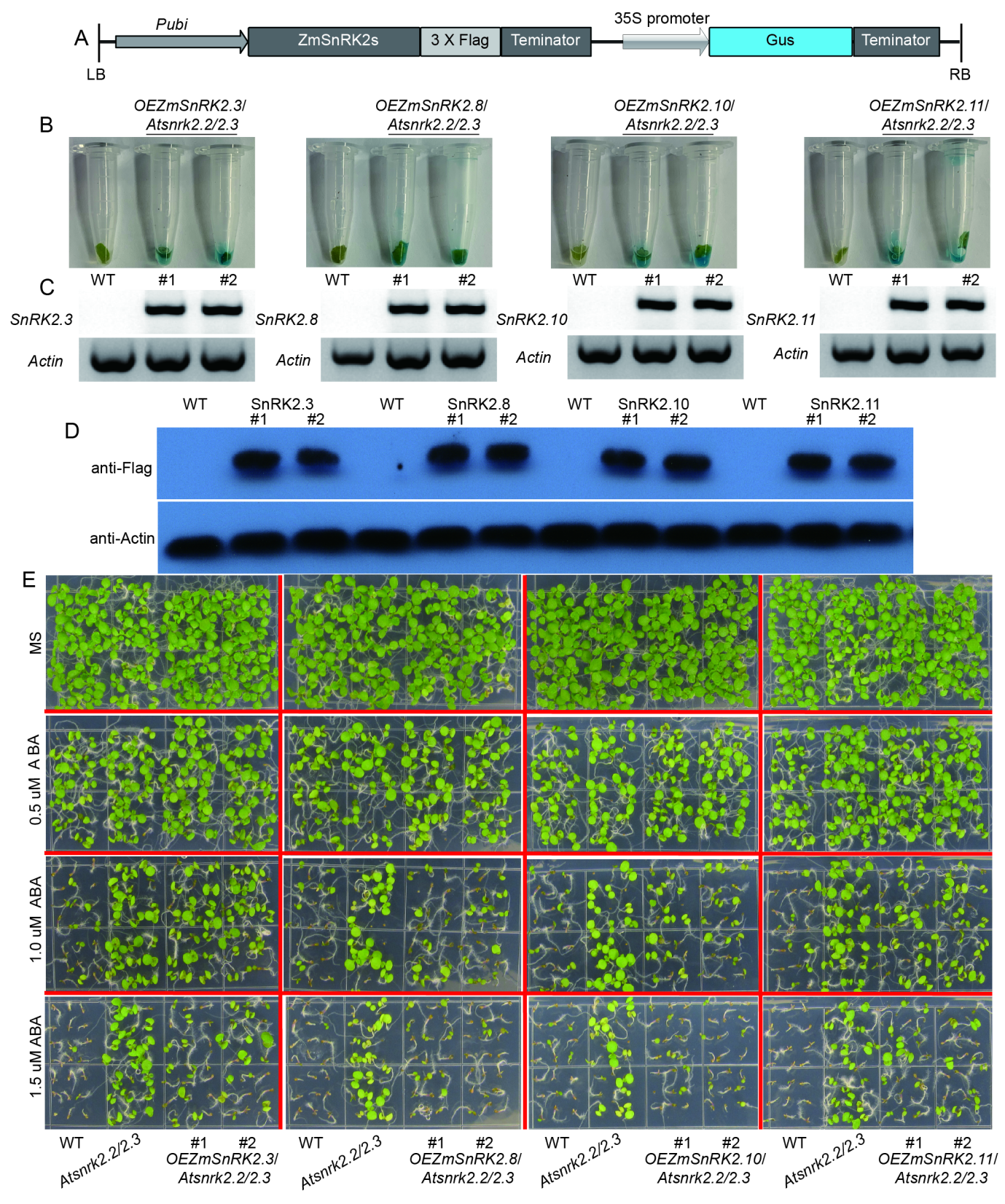


Fig. S6 Overexpression of *ZmSnRK2.8* and *ZmSnRK2.10*, not *ZmSnRK2.3* and *ZmSnRK2.11*, could rescue the ABA sensitivity of *snrk2.2/2.3*. (A) The structure of vector used in transgene analysis. (B) Identification of positive transgenic plants by GUS staining. (C) Identification of positive transgenic plants by PCR. (D) Identification of positive transgenic plants by immunoblotting. (E) Phenotype of transgenic plants after growth on MS media supplemented with or without ABA for 10 days.


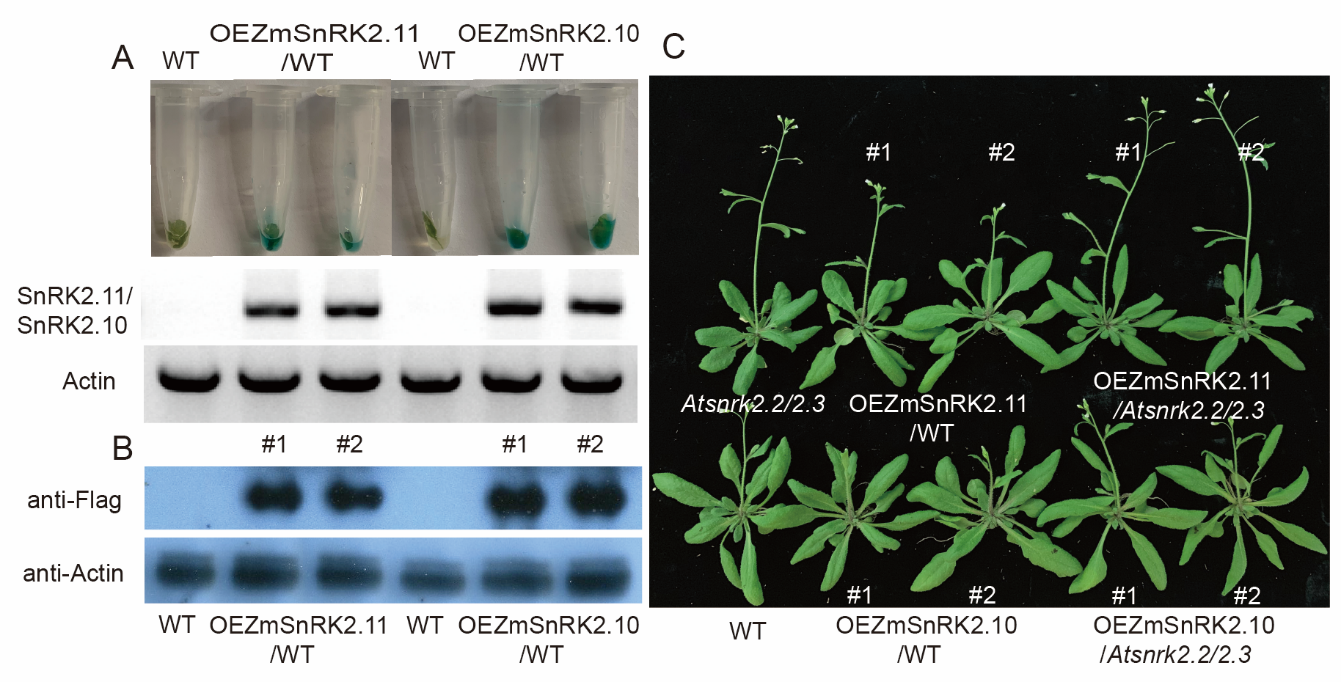


Fig. S7 Overexpression of *ZmSnRK2.10* leads to delayed flowering time in Arabidopsis. (A) Identification of positive transgenic plants by PCR. (B) Identification of positive transgenic plants by immunoblotting. (C) Flowering phenotype of the WT, *Atsnrk2.2/2.3* and transgenic plants. The photograph was taken 35 d days after growth under LD conditions.


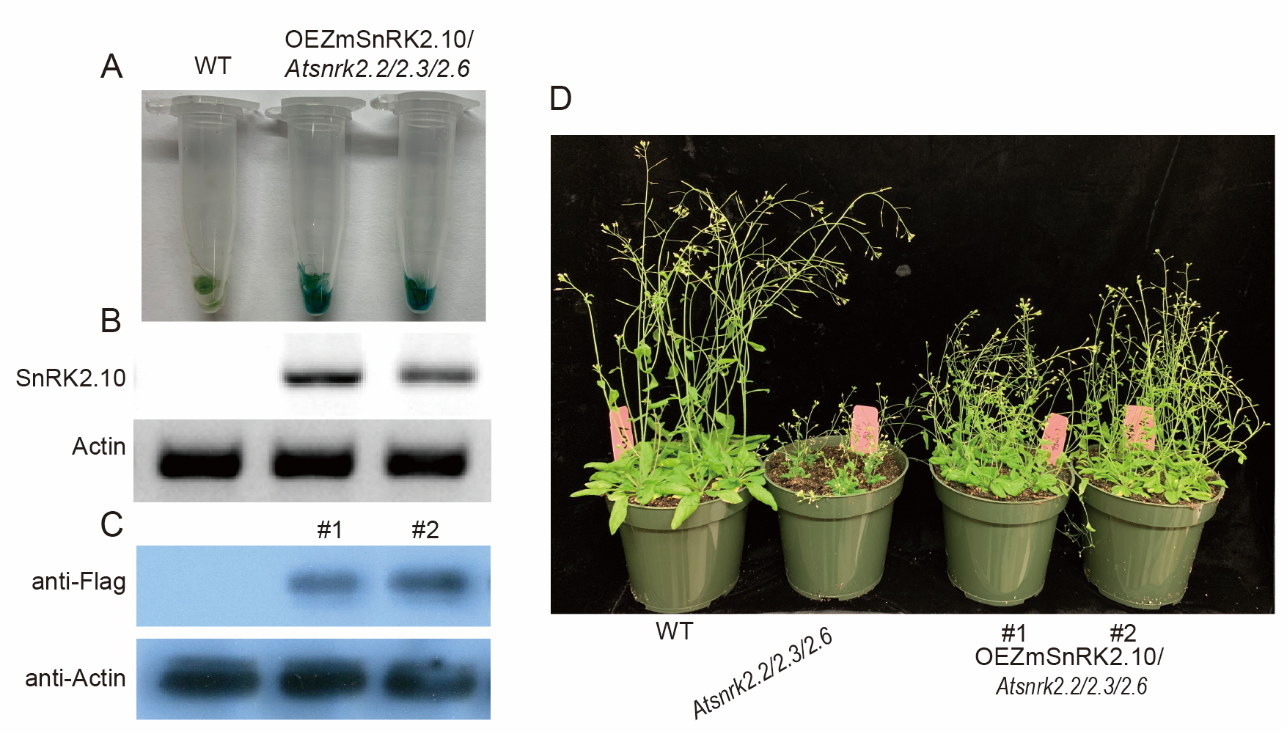


Fig. S8 Overexpression of ZmSnRK2.10 in snrk2.2/2.3/2.6 Arabidopsis can partially rescue the phenotype. (A) Identification of positive transgenic plants by GUS staining. (B) Identification of positive transgenic plants by PCR. (C) Identification of positive transgenic plants by immunoblotting. (D) The phenotype of WT, *Atsnrk2.2/2.3/2.6*, and *OEZmSnRK2.10*/*Atsnrk2.2/2.3/2.6* after growth for 40 days under LD conditions.

Additional file 1

Table S1 Characteristics of the SnRK2 gene family in maize.

|  | Number | Gene ID | Chr# | Extron | Protein length | Isoelectric  Point (pI) | Protein Mol.  Weight (kDa) | Domain  size | Start & End Position of Each Gene on genome | cDNA  length | | CDS  length | |  |
| --- | --- | --- | --- | --- | --- | --- | --- | --- | --- | --- | --- | --- | --- | --- |
|  | ZmSnRK2.1 | Zm00001d047220 | 9 | 9 | 354 | 5.158 | 40.092 | 4-272 | 122717205-122721608 | 1462 | | 1065 | |  |
|  | ZmSnRK2.2 | Zm00001d022179 | 7 | 9 | 339 | 5.230 | 38.561 | 4-260 | 171984109-171989937 | 1614 | | 1020 | |  |
|  | ZmSnRK2.3 | Zm00001d029975 | 1 | 9 | 333 | 5.356 | 37.841 | 5-261 | 9711273-97116269 | 1501 | | 1002 | |  |
|  | ZmSnRK2.4 | Zm00001d042695 | 3 | 9 | 361 | 6.277 | 42.226 | 4-260 | 177276266-177281835 | 1702 | | 1086 | |  |
|  | ZmSnRK2.5 | Zm00001d026690 | 10 | 2 | 363 | 6.732 | 42.838 | 4-260 | 150050330-150051860 | 1463 | | 1092 | |  |
|  | ZmSnRK2.6 | Zm00001d050723 | 4 | 9 | 364 | 6.027 | 41.786 | 4-260 | 118811903-118822922 | 1626 | | 1095 | |  |
|  | ZmSnRK2.7 | Zm00001d003659 | 2 | 9 | 356 | 6.431 | 40.690 | 4-260 | 52839413-52844416 | 1545 | | 1071 | |  |
|  | ZmSnRK2.8 | Zm00001d034161 | 1 | 9 | 366 | 4.580 | 41.371 | 28-284 | 285417447-285425538 | 1729 | | 1101 | |  |
|  | ZmSnRK2.9 | Zm00001d033339 | 1 | 8 | 364 | 4.568 | 40.860 | 23-279 | 259579890-259583075 | 1803 | | 1095 | |  |
|  | ZmSnRK2.10 | Zm00001d013736 | 5 | 8 | 362 | 4.567 | 40.804 | 23-279 | 18918646-18921698 | 2028 | | 1089 | |  |
|  | ZmSnRK2.11 | Zm00001d038326 | 6 | 9 | 359 | 5.858 | 41.511 | 4-260 | 154559425-154564538 | | 1636 | | 1080 | |
|  | ZmSnRK2.12 | Zm00001d013201 | 5 | 9 | 366 | 4.580 | 41.440 | 28-284 | 6396505-6403575 | 1635 | | 1101 | |  |
|  | ZmSnRK2.13 | Zm00001d016783 | 5 | 11 | 364 | 6.100 | 41.850 | 4-260 | 175764979-175770061 | 1507 | | 1095 | |  |
|  | ZmSnRK2.14 | Zm00001d012263 | 8 | 9 | 355 | 6.632 | 41.391 | 4-260 | 171421196-171427134 | 1722 | | 1068 | |  |

The Gene ID means the accession numbers of the protein at the Genome Database of MaizeGDB for B73.

Table S2 Primers used in this article.

| Name | Sequence (5′-3′) |
| --- | --- |
| Primers used for real time PCR | |
| AtACTIN-F | TAACAGGGAGAAGATGACTCAGATCA |
| AtACTIN-R | AAGATCAAGACGAAGGTAAGCATGAG |
| ZmActin-F | TCACTACGACTGCCGAGCGAG |
| ZmActin-R | GAGCCACCACTGAGGACAACATTAC |
| ZmSnRK2.1-QF | GACTACGTTCGCGTTACAATGG |
| ZmSnRK2.1-QR | CGATGGGCAGATTCTTGAGG |
| ZmSnRK2.2-QF | ATCTGCTGTCTCGGATCTTTGTG |
| ZmSnRK2.2-QR | TTCATCTGGTACTCGTCGGTCA |
| ZmSnRK2.3-QF | AGATATTCGTTGCCGATCCTTC |
| ZmSnRK2.3-QR | GCGTCCTTGTAGTTGGCCTTC |
| ZmSnRK2.4-QF | TTGCCCGTATCTTTGTTGCC |
| ZmSnRK2.4-QR | TCCGTGAGTTCCCGTGGTAG |
| ZmSnRK2.5-QF | CCGACGAAGCAGAGCAACA |
| ZmSnRK2.5-QR | ATGTCGAACTCTCCGCTGGC |
| ZmSnRK2.6-QF | TTGGGCAGAGGAAGAAGAGCA |
| ZmSnRK2.6-QR | GCTGGCATGTACTTGCTTCACC |
| ZmSnRK2.7-QF | GTGTCGCAAGACTGCAAGGAG |
| ZmSnRK2.7-QR | GCTCTCTGGGCAGGTTCTTCA |
| ZmSnRK2.8-QF | CCACCATACCACCTGCCTGTT |
| ZmSnRK2.8-QR | CAATCTCACCGCTGCTGTCAA |
| ZmSnRK2.9-QF | GGACCCAGACGAGCCTAAGAA |
| ZmSnRK2.9-QR | CTGGAGATTAGGTCCTGGCATT |
| ZmSnRK2.10-QF | ATGACAGCACGATGAGCAAGC |
| ZmSnRK2.10-QR | CGGTATCTGAGTCCAGGTCGTC |
| ZmSnRK2.11-QF | GGCTATGGTTACAGTGAAGAATGC |
| ZmSnRK2.11-QR | CGAACCTCCCTAACTGTCTTATCAC |
| ZmSnRK2.12-QF | AGCATGAGCAACCAATATGAGGA |
| ZmSnRK2.12-QR | CGAGAACAGGCAGGTGGTATG |
| ZmSnRK2.13-QF | CTCCTGGCTCGGATCTTCGT |
| ZmSnRK2.13-QR | TTCTTGTAGTACTTCGCTTGCGC |
| ZmSnRK2.14-QF | TATTGCCCGTATCTTTGTTGCC |
| ZmSnRK2.14-QR | TCTCCGTGAGTTCCCGTGGT |
| Primers used to generate DNA/RNA constructs | |
| ZmSnRK2.1-2300F | ggagaggacagggtacccgggATGGAGCGGTACGAGGTGATC |
| ZmSnRK2.1-2300R | ggtactagtgtcgactctagaCACTGCACAAACGAAGTCGCC |
| ZmSnRK2.2-2300F | ggagaggacagggtacccgggATGGAGAGGTACGAGGTGATCAA |
| ZmSnRK2.2-2300R | ggtactagtgtcgactctagaCAAGGCACATACAAAGTCGCC |
| ZmSnRK2.3-2300F | ggagaggacagggtacccgggATGGAGGAGAGGTACGAGGCG |
| ZmSnRK2.3-2300R | ggtactagtgtcgactctagaGTAGGTGTCATCGGCGTCTGC |
| ZmSnRK2.4-2300F | ggagaggacagggtacccgggATGGACAAGTACGAGGCGGTG |
| ZmSnRK2.4-2300R | ggtactagtgtcgactctagaGCTTTCATATGTGAAGAGCGTCC |
| ZmSnRK2.5-2300F | ggagaggacagggtacccgggATGGACAAGTACGAGCCCGTT |
| ZmSnRK2.5-2300R | ggtactagtgtcgactctagaGATTTGGAGTCTGCTCATAATCATG |
| ZmSnRK2.6-2300F | ggagaggacagggtacccgggATGGAGAGGTACGAACTGCTCAA |
| ZmSnRK2.6-2300R | ggtactagtgtcgactctagaTTTTATTAGGTGTCGAAAGTCCCC |
| ZmSnRK2.7-2300F | ggagaggacagggtacccgggATGGAGAAGTACGAGCTGCTGAA |
| ZmSnRK2.7-2300R | ggtactagtgtcgactctagaGCAGCTGAGATGAAACTCCCC |
| ZmSnRK2.8-2300F | ggagaggacagggtacccgggATGGCAGGGCCGGCGCCG |
| ZmSnRK2.8-2300R | ggtactagtgtcgactctagaCATTGCGTACACAATCTCACCG |
| ZmSnRK2.9-2300F | ggagaggacagggtacccgggATGGACCGGGCGGCGCTC |
| ZmSnRK2.9-2300R | ggtactagtgtcgactctagaCATAGCGTACACGATCTCCCCG |
| ZmSnRK2.10-2300F | ggagaggacagggtacccgggATGGACCGGGCGGCGCTC |
| ZmSnRK2.10-2300R | ggtactagtgtcgactctagaAATAGCATACACGATTTCCCCAC |
| ZmSnRK2.5-2300F | ggagaggacagggtacccgggATGGACAAGTACGAGCCCGTT |
| ZmSnRK2.5-2300R | ggtactagtgtcgactctagaGATTTGGAGTCTGCTCATAATCATG |
| ZmSnRK2.6-2300F | ggagaggacagggtacccgggATGGAGAGGTACGAACTGCTCAA |
| ZmSnRK2.6-2300R | ggtactagtgtcgactctagaTTTTATTAGGTGTCGAAAGTCCCC |
| ZmSnRK2.7-2300F | ggagaggacagggtacccgggATGGAGAAGTACGAGCTGCTGAA |
| ZmSnRK2.7-2300R | ggtactagtgtcgactctagaGCAGCTGAGATGAAACTCCCC |
| ZmSnRK2.8-2300F | ggagaggacagggtacccgggATGGCAGGGCCGGCGCCG |
| ZmSnRK2.8-2300R | ggtactagtgtcgactctagaCATTGCGTACACAATCTCACCG |
| ZmSnRK2.9-2300F | ggagaggacagggtacccgggATGGACCGGGCGGCGCTC |
| ZmSnRK2.9-2300R | ggtactagtgtcgactctagaCATAGCGTACACGATCTCCCCG |
| ZmSnRK2.10-2300F | ggagaggacagggtacccgggATGGACCGGGCGGCGCTC |
| ZmSnRK2.10-2300R | ggtactagtgtcgactctagaAATAGCATACACGATTTCCCCAC |
| ZmSnRK2.11-2300F | ggagaggacagggtacccgggATGGACAAGTACGAGGCTGTTCG |
| ZmSnRK2.11-2300R | ggtactagtgtcgactctagaGATGTGCAGTGAGGTCATATCCAG |
| ZmSnRK2.12-2300F | ggagaggacagggtacccgggATGGCAGGGCCGGCGCCG |
| ZmSnRK2.12-2300R | ggtactagtgtcgactctagaCATTGCGTATACAATCTCACCGC |
| ZmSnRK2.13-2300F | ggagaggacagggtacccgggATGGAGAAGTACGAGCTGCTCAA |
| ZmSnRK2.13-2300R | ggtactagtgtcgactctagaTTTTAGGAGGTGTTGAAAATCCCC |
| ZmSnRK2.3-1305F | acgggggactctagaggatccATGGAGGAGAGGTACGAGGCG |
| ZmSnRK2.3-1305R | agtccatggagatctaagcttGTAGGTGTCATCGGCGTCTGC |
| ZmSnRK2.8-1305F | acgggggactctagaggatccATGGCAGGGCCGGCGCCG |
| ZmSnRK2.8-1305R | agtccatggagatctaagcttCATTGCGTACACAATCTCACCG |
| ZmSnRK2.10-1305F | acgggggactctagaggatccATGGACCGGGCGGCGCTC |
| ZmSnRK2.10-1305R | agtccatggagatctaagcttAATAGCATACACGATTTCCCCAC |
| ZmSnRK2.11-1305F | acgggggactctagaggatccATGGACAAGTACGAGGCTGTTCG |
| ZmSnRK2.11-1305R | agtccatggagatctaagcttGATGTGCAGTGAGGTCATATCCAG |
| ZmSnRK2.3-FlagF | gacgatgacgataagggatccATGGAGGAGAGGTACGAGGCG |
| ZmSnRK2.3-FlagR | cgatcggggaaattcgagctcGTAGGTGTCATCGGCGTCTGC |
| ZmSnRK2.8-FlagF | gacgatgacgataagggatccATGGCAGGGCCGGCGCCG |
| ZmSnRK2.8-FlagR | cgatcggggaaattcgagctcATTGCGTACACAATCTCACC |
| ZmSnRK2.10-FlagF | gacgatgacgataagggatccATGGACCGGGCGGCGCTC |
| ZmSnRK2.10-FlagR | cgatcggggaaattcgagctcAATAGCATACACGATTTCCCCAC |
| ZmSnRK2.11-FlagF | gacgatgacgataagggatccATGGACAAGTACGAGGCTGTTCG |
| ZmSnRK2.11-FlagR | cgatcggggaaattcgagctcGATGTGCAGTGAGGTCATATCCAG |
